# Solution and Solid-State Behavior of Amphiphilic ABA Triblock Copolymers of Poly(acrylic acid-*stat*-styrene)-*block*-poly(butyl acrylate)-*block*-poly(acrylic acid-*stat*-styrene)

**DOI:** 10.1021/acs.macromol.2c01299

**Published:** 2022-10-28

**Authors:** Thomas J. Neal, Robert D. Bradley, Martin W. Murray, Neal S. J. Williams, Simon N. Emmett, Anthony J. Ryan, Sebastian G. Spain, Oleksandr O. Mykhaylyk

**Affiliations:** † Department of Chemistry, The University of Sheffield, Dainton Building, Sheffield, South YourkshireS3 7HF, U.K.; ‡ AkzoNobel Decorative Paints, Wexham Road, Slough, BerkshireSL2 5DS, U.K.

## Abstract

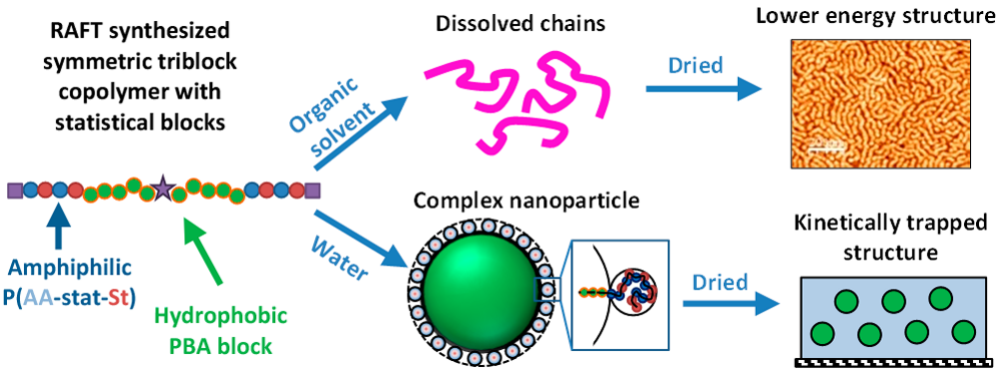

A combination of statistical and triblock copolymer properties is explored to produce stable aqueous polymer dispersions suitable for the film formation. In order to perform an extensive structural characterization of the products in the dissolved, dispersed, and solid states, a wide range of symmetrical poly(acrylic acid-*stat*-styrene)_*x*_-*block*-poly(butyl acrylate)_*y*_-*block*-poly(acrylic acid-*stat*-styrene)_*x*_, poly(AA-*st*-St)_*x*_-*b*-PBA_*y*_-*b*-poly(AA-*st*-St)_*x*_, (*x* = 56, 108 and 140, *y* = 100–750; the AA:St molar ratio is 42:58) triblock copolymers were synthesized by reversible addition–fragmentation chain transfer (RAFT) solution polymerization using a bifunctional symmetrical RAFT agent. It is demonstrated that the amphiphilic statistical outer blocks can provide sufficient stabilization to largely hydrophobic particles in aqueous dispersions. Such a molecular design provides an advantage over copolymers composed only of homoblocks, as a simple variation of the statistical block component ratio provides an efficient way to control the hydrophilicity of the stabilizer block, which ultimately affects the copolymer morphology in solutions and solid films. It was found by small-angle X-ray scattering (SAXS) that the copolymers behaved as dissolved chains in methylethylketone (MEK) but self-assembled in water into stable and well-defined spherical particles that increased in size with the length of the hydrophobic PBA block. These particles possessed an additional particulate surface structure formed by the statistical copolymer stabilizer block, which self-folded through the hydrophobic interactions between the styrene units. SAXS and atomic force microscopy showed that the copolymer films cast from the MEK solutions formed structures predicted by self-consistent field theory for symmetrical triblock copolymers, while the aqueous dispersions formed structural morphologies similar to a close-packed spheres, as would be expected for copolymer particles trapped kinetically due to the restricted movement of the blocks in the initial aqueous dispersion. A strong correlation between the structural morphology and mechanical properties of the films was observed. It was found that the properties of the solvent cast films were highly dependent on the ratios of the hard [poly(AA-*st*-St)] and soft (PBA) blocks, while the aqueous cast films did not show such a dependence. The continuous phase of hard blocks, always formed in the case of the aqueous cast films, produced films with a higher elastic modulus and a lower extension-to-break in a comparison with the solvent-cast films.

## Introduction

The self-assembly of block copolymers in both solution and in the bulk has been widely researched over many decades.^1−8^ In solution, these systems are known to form various morphologies, such as spheres,^9−17^ worms,^18,19^ and vesicles,^20,21^ and can be used for a variety of applications such as for drug delivery^22^ or as thickening agents.^23^ In the solid state, block copolymers self-assemble to form gyroid, hexagonally packed cylindrical, body-centered cubic (BCC), and lamellae structures that are often used in industry (e.g., as thermoplastic elastomers).^8,24^ It is widely known that the self-assembly of block copolymers is dictated by the length and chemical properties of each block.^25^ Using a combination of blocks with a high glass transition temperature (*T*_g_) and blocks with a low *T*_g_, one can create materials that are both tough and flexible.^26−28^ The family of synthetic rubbers known as Kratons is an example of such systems. Kratons are ABA triblock copolymers, where the A block has a high *T*_g_ (hard block) such as polystyrene and the B block has a low *T*_g_ (soft block) such as polybutadiene. In the solid state, these triblocks self-assemble into hard spherical domains of polystyrene that are covalently linked by a matrix of rubbery polybutadiene. Due to the physically cross-linked structure of the copolymer, the resulting film has very favorable mechanical properties (i.e., strong and flexible).

Although copolymers such as Kraton provide a useful “gold-standard” for block copolymer materials in the bulk, they are not suitable for use as coatings, as they would need to be cast from solutions in harmful organic solvents. For this reason, a large amount of block copolymer research is focused on water-dispersed systems.^29−31^ In these systems, a purely hydrophilic stabilizer block is often used, as it provides good colloidal stability within the dispersion through either steric^32^ or electrostatic^30^ stabilization. However, in the solid phase this leads to large regions of hydrophilic groups, which may be disadvantageous for some applications, such as water-resistant coatings, e.g. gloss paints. For example, it was recently reported^27^ that poly(acrylic acid)-*b*-poly(butyl acrylate) (PAA-*b*-PBA) diblock copolymer particles synthesized by reversible addition–fragmentation chain transfer (RAFT) aqueous emulsion polymerization-induced self-assembly could be used to form tough and transparent films. As the latex dries, the core–shell structure of the particle is maintained because the hard acrylic acid shell prevents the interdiffusion of the soft butyl acrylate cores. This results in a honeycomb-like network of cores separated by a a percolating network of poly(acrylic acid) . However, despite some benefits of a percolating network of acrylic acid in regards to the film toughness, this continuous network of hydrophilic units offers a potential path for the transport of water through the copolymer coating. Furthermore, the more thermodynamically stable conformation (spherical regions of poly(acrylic acid) in a continuous matrix of poly(butyl acrylate)) is only achieved once the polymer is annealed above the *T*_g_ of poly(acrylic acid).^27^ Rieger and co-workers developed this work further by integrating high-*T*_g_ polystyrene-core nanofibers into a dispersion of PAA-*b*-PBA spherical nanoparticles.^26^ The anisotropic high-*T*_g_ nanofibers act as reinforcing fillers once the acrylic aqueous dispersion dries onto a film and are responsible for both the increased stiffness of the material and maintaining high extensibility.^26^

Similar studies have been performed on more complex multiblock copolymers. Qiao et al. reported the synthesis of symmetrical ABCBA pentablock copolymers via solvent-free aqueous RAFT emulsion polymerization using a symmetrical RAFT agent, where the copolymers self-assembled into a three-layer core–shell spherical structure during the polymerization.^33^ Here, the particles were also stabilized by a block of poly(acrylic acid); thus, when the copolymer dispersion was dried, a similar structure was observed in the copolymer film compared to that previously observed by Chenal et al., i.e., a percolating network of poly(acrylic acid).^33^

Compared to block copolymers, the self-assembly of statistical copolymers is relatively understudied. However, there has been a recent resurgence in interest in these systems, as they are know to self-assemble into useful nano-objects such as spheres,^34−36^ rods or worms,^37−39^ vesicles,^36,40^ and “bowl-like” morphologies^41^ and can also be tuned to form single-chain nanoparticles (SCNPs).^42−50^ Furthermore, the self-assembly behavior of statistical copolymers is very predictable on the basis of the copolymer composition and the solvophobic nature of the copolymer units.^42,51^ It was recently demonstrated that charge-stabilized amphiphilic statistical copolymers undergo spontaneous self-assembly driven by hydrophobic interactions to form well-defined spherical particles, where the relationship between particle size and the copolymer composition can be described using a particle surface charge (PSC) model.^42,51^

Statistical copolymers can form a range of different morphologies. It was recently reported that amphiphilic statistical copolymers formed “multicompartment” nanostructures in solution by orthogonal folding.^49^ The copolymers studied therein were composed of two distinct statistical blocks of statistical copolymers [i.e., P(A-*st*-C)-*b*-P(B-*st*-C) ,where A and B are different hydrophobic monomers and C is a hydrophilic monomer]. It was reported that the two distinct blocks will “self-sort” or phase-separate. This phase separation, in addition to the hydrophobic interactions that drive the self-assembly into micelle-like structures in which the hydrophilic component remains at the water interface and the hydrophobic components aggregate to form a particle core, leads to these complex multicompartmented structures.^49^ In addition, it was also examined how amphiphilic crystalline statistical copolymer dispersions assemble in the bulk once the dispersion is dried.^52^ It was found that statistical copolymers with a low hydrophobe content (<50 mol %) self-assembled into spherical micelles, whereas copolymers with higher hydrophobe content (>50 mol %) self-assembled into vesicles. Furthermore, after the removal of the solvent, the bulk structures observed for both the spherical micelle and the vesicle dispersions were vastly different, forming a BCC structure and a lamellar structure, respectively.^52^

In this study, to challenge whether a purely hydrophilic stabilizer is required to stabilize a hydrophobic particle, properties of both statistical and block copolymers are explored in combination by synthesizing block copolymers that can be dispersed in water using an amphiphilic statistical stabilizer block. Furthermore, it is demonstrated that an amphiphilic statistical copolymer block can provide sufficient stabilization to a largely hydrophobic particle.

It is found herein that the proposed copolymer systems undergo a twofold self-assembly process when dispersed in water: (i) self-assembly of the hydrophobic core and (ii) self-assembly within the amphiphilic stabilizer block. Specifically, it is demonstrated that the poly(acrylic acid-*stat*-styrene)-*b*-poly(butyl acrylate)-*b*-poly(acrylic acid-*stat*-styrene) [P(AA-*st*-St)_*x*_-*b*-PBA_*y*_-*b*-P(AA-*st*-St)_*x*_ or ABA] triblock copolymer composition allows a wide range of copolymer properties to be varied. The solution structures of these copolymers were assessed in both solvent and water using small-angle X-ray scattering (SAXS) to observe how they affected the bulk copolymer structure when cast as films. The resultant mechanical properties of both the solvent-cast films and the water-cast films were also assessed and linked to structural morphologies.

## Experimental Section

### Materials

Butyl acrylate (BA, 99%), acrylic acid (AA, 99%), styrene (St, 99%), isopropanol (IPA, 99.9%), methyl ethyl ketone (MEK, 99.9%), ammonia (NH_3_, 25%), glacial acetic acid (99.85%), high-performance liquid chromatography-grade tetrahydrofuran (THF), and deuterated dimethyl sulfoxide (*d*_6_-DMSO) were purchased from Sigma-Aldrich (Gillingham, UK). *S*,*S*-Dibenzyl trithiocarbonate (DBzTTC) was purchased from Boron Molecular (Raleigh, NC). All materials were used as received unless otherwise stated. Deionized water was obtained from an ELGA Elgastat Option 3A water purifier (High Wycombe, UK). All materials were used as received unless stated otherwise in the text.

### Synthesis of P(AA-*st*-St) Macro-CTA via RAFT Solution Polymerization

**Figure 1:**
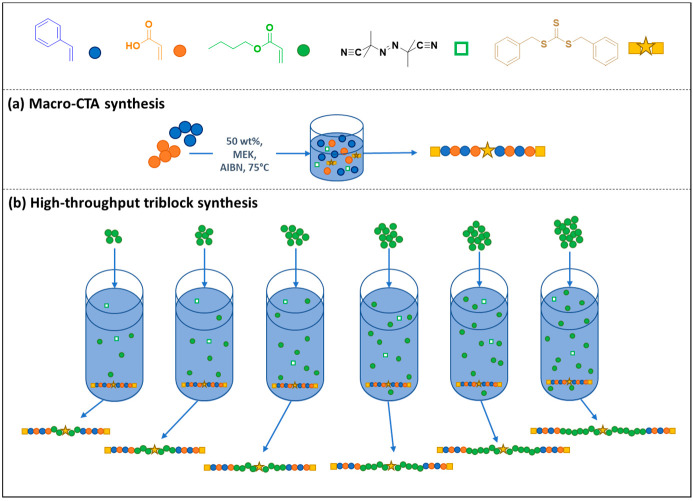
RAFT polymerization protocol used to produce the sets of different P(AA-*st*-St)_*x*_-*b*-PBA_*y*/2_-DBzTTC-PBA_*y*/2_-*b*-P(AA-*st*-St)_*x*_ (A_*x*_B_*y*_A_*x*_) triblock copolymers, namely (a) the P(AA-*st*-St)_*x*_-DBzTTC-P(AA-*st*-St)_*x*_ (A_*x*_-DBzTTC-A_*x*_) macro-CTA synthesis used to prepare A_56_-DBzTTC-A_56_ (A_56_A_56_), A_108_A_108_, and A_140_A_140_ (Table 1) and (b) the high-throughput synthesis of the triblock copolymers A_56_B_100_-_750_A_56_, A_112_B_100_-_750_A_112_, and A_140_B_100_-_750_A_140_ (Table 2) using a Chemspeed high-throughput robot.

Three P(AA-*st*-St)_*x*_-DBzTTC-P(AA-*st*-St)_*x*_ (A_*x*_-DBzTTC-A_*x*_ or, for the sake of simplicity, A_*x*_A_*x*_, which should not be confused with the AA abbreviation used for acrylic acid) macro-CTAs with a targeted AA/St molar ratio of 50:50 but different degrees of polymerization (DP) (*x* = DP/2 = 56, 108, and 140) were synthesized (Figure 1a and Table 1). The protocol describes the quantities used to synthesize A_56_-DBzTTC-A_56_ (A_56_A_56_); a full table of quantities can be found in the Supporting Information (Table S1). AA (33.9 g, 0.471 mol), St (49.1 g, 0.471 mol), AIBN (0.186 g, 0.00114 mol), and DBzTTC (1.65 g, 0.00568 mol) were dissolved in MEK (105 mL). The solution was degassed with bubbling N_2_ for 60 min and then heated to 80 °C to initiate polymerization. This polymerization was quenched in air after 7 h at a monomer conversion of 68%. The initial P(AA-*st*-St) macro-CTA was purified by multiple precipitations from hexane, and the product was collected as a solid yellow powder.

**Table 1:** Compositions and Molar Masses of Macro-CTAs Used for the P(AA-*st*-St)-*b*-PBA-*b*-P(AA-*st*-St) (ABA) Triblock Copolymer Synthesis

		NMR		SEC^c^		
copolymer	conversion (%)	DP of copolymer^a^	mole fraction of AA^b^	*M*_n_ (kDa)	*M*_w_ (kDa)	*M*_w_/*M*_n_
A_56_A_56_	68	112 (56 + 56)	0.42	9.1	11.4	1.26
A_108_A_108_	58	216 (108 + 108)	0.42	15.9	19.8	1.24
A_140_A_140_	56	280 (140 + 140)	0.42	19.2	24.5	1.28

a The total DP of both A blocks used to synthesize the ABA triblock, where the A blocks will be half the DP of the respective macro-CTA. The DPs were calculated using the ratio of the integrals from the RAFT chain end and the copolymer backbone.

b The copolymer composition was calculated by examining the respective integrals in the ^1^H NMR spectrum in DMSO.

c SEC measurements were performed using a THF eluent containing 1% v/v acetic acid against PSt standards.

### Parallel Syntheses of Triblock Copolymers via RAFT Solution Polymerization

Specific quantities (Table S1) of the purified P(AA-*st-*St) macro-CTA were added to eight reaction vessels of the Chemspeed Autoplant A100 high-throughput robot (Chemspeed Technologies, Switzerland). The macro-CTA was then dissolved in MEK at 50 °C. Once the macro-CTAs were fully dissolved, the solution was cooled to ambient temperature, and BA (5.00 g, 35.2 mmol) was added. Quantities of an AIBN solution (14 mg mL^–1^) in MEK were added to the eight reaction vessels and then a stream of nitrogen was blown through all the reaction vessels for 30 min in order to expel any oxygen. The mixtures were then heated to 80 °C and stirred with an overhead anchor stirrer for 8 h. The reaction mixtures were left to cool to ambient temperature overnight and decanted into 100 mL sample pots (Figure 1b). These copolymers were either precipitated into hexane, dried, and redispersed in water or dried as films straight from the reaction solution. Since butyl acrylate is a volatile organic compound, it is assumed that once the butyl acrylate dries there is no monomer present in the final copolymer film.

### ^1^H Nuclear Magnetic Resonance (NMR) Spectroscopy

^1^H NMR spectra were recorded on a Bruker AV1-400 or AV3HD-400 MHz spectrometer in *d*_6_-DMSO. These spectra were analyzed using Bruker Topspin software (version 3.5pl7), and chemical shifts are reported relative to a residual solvent peak.

### Size Exclusion Chromatography/Advanced Polymer Chromatography (SEC/APC)

Molar mass distributions of the triblock copolymers were determined by SEC using THF with 1% v/v acetic acid as the eluent. Measurements were performed on a Waters ACQUITY APC system equipped with a refractive index detector. Separations were carried out using a set of 150 mm XT columns (45, 125, and 450 Å) at a flow rate of 1.0 mL min^–1^. All the samples were filtered through 0.2 μm PTFE membrane filters prior to analysis and measured relative to low-dispersity polystyrene (PSt) standards with peak molar masses ranging from 580 to 8 000 000 Da.

### Small-Angle X-ray Scattering (SAXS) Measurements

SAXS samples were prepared by diluting the investigated polymer solution or dispersions with the appropriate solvent (MEK or water) to the desired concentration (e.g., 1%, 5% or 20% w/w). SAXS patterns were collected using laboratory SAXS instruments, either a Bruker AXS Nanostar instrument equipped with a two-dimensional (2D) Hi-STAR multiwire gas detector and modified with Xenocs GeniX 3D X-ray source (Cu Kα radiation, X-ray wavelength λ = 1.54 Å) and motorized collimating scatterless slits or a Xenocs Xeuss 2.0 laboratory beamline equipped with a 2D Dectris Pilatus 1 M detector and an Excillum liquid gallium MetalJet X-ray source (λ = 1.34 Å). The scattering patterns of the liquids were collected in a 2 mm glass capillary either positioned in a capillary holder or integrated in a flow-through cell to allow scattering patterns to be collected under vacuum. The patterns were collected over a scattering vector length range of 0.008 Å^–1^ < *q* < 0.16 Å^–1^, where  and θ is a half the scattering angle. One-dimensional (1D) scattering curves were obtained by azimuthal binning and averaging the corresponding two-dimensional scattering patterns using software packages supplied with the SAXS instruments. Calibration, background subtraction, and further analysis of the 1D SAXS data were performed using Irena SAS macros for Igor Pro.^53^

### Grazing-Incident Small-Angle X-ray Scattering (GISAXS) Measurements

The grazing-incident SAXS samples were prepared by drop-casting approximately 100 μL of the 20% w/w aqueous dispersion onto a clear ruby-grade mica disc 25 μm thick with a 15 mm diameter (Attwater group, UK). The dispersion was left to dry for 1 week. GISAXS measurements were performed using the Xenocs Xeuss 2.0 laboratory beamline. Time-resolved GISAXS was performed on block copolymer films during annealing in air at elevated temperatures (150 °C). To perform the measurements, the films were cast on to a mica substrate that was then mounted on a modified Linkam heating stage attached to a goniometer head. The measurements were performed at a critical angle, which was established prior to data acquisition. 1D scattering curves were obtained by azimuthal binning, taking into account the incident angle, and averaging the corresponding 2D scattering patterns using the software package supplied with the laboratory SAXS beamline.

### Atomic Force Microscopy (AFM)

The investigated triblock copolymers were either drop-cast directly onto small metal AFM plates or drop-cast onto a silicone-coated dry-release film with a 300 μm thickness (Avery Dennison) and then attached to an AFM plate using double-sided tape. AFM height (topographic) images were collected using the ScanAsyst PeakForce tapping mode on a Bruker MultiMode atomic force microscope. A 2 nm silicon ScanAsyst-Air cantilever was used to perform the measurements. The WsXM 4.0 software was used for the image analysis, including measurements of the copolymer phase-separation length scale.^54^

### Mechanical Testing

Triblock copolymer films were cast in plastic molds and left to dry under ambient conditions for one week. The films were then removed from the molds and cut into strips with a width of 7 mm and a length greater than 13 mm (the set gap). The individual thicknesses were measured using a micrometer, and both the extension-to-break and the Young’s modulus were measured at ambient conditions using an Instron 5500R instrument. All the triblock copolymer films cast from MEK were very uniform and showed no signs of either dewetting from the substrate or forming bubbles within the film. However, the copolymer films cast from water showed large amounts of dewetting from the plastic mold, and the formation of bubbles was observed during the drying process; in some cases, these two factors made it difficult to prepare uniform films. The observed dewetting is likely to be due to incompatibilities between the water and the substrate,^55,56^ whereas the formation and stabilization of bubbles within the aqueous dispersions is a result of the amphiphilic nature of the copolymers and their ability to behave like surfactants.^56^

## Results and Discussion

### Synthesis and Characterization of Copolymers

A set of P(AA-*st*-St)_*x*_-*b*-PBA_*y*/2_-DBzTTC-PBA_*y*/2_-*b*-P(AA-*st*-St)_*x*_ (A_*x*_B_*y*_A_*x*_) triblock copolymers was synthesized using RAFT copolymerization. A symmetrical bifunctional RAFT agent (DBzTTC) was used in order to achieve symmetrical triblock copolymers in two steps: (i) synthesis of a poly(acrylic acid-*stat*-styrene) macro-CTA with a midchain trithiocarbonate (Figure 1a) and (ii) chain extension “from the middle” with butyl acrylate to yield the desired A_*x*_B_*y*_A_*x*_ triblock copolymer (Figure 1b). First, a series of statistical copolymer macro-CTAs that would form the “A” blocks of the triblock copolymers were synthesized via RAFT copolymerization of styrene and acrylic acid at 50% w/w in MEK (Figure 1a). The resulting copolymers were isolated by precipitation from hexane.

Since both PSt and PAA are reported to have high glass transition temperatures (*T*_g_ ∼ 100 °C),^57,58^ they will be denoted as the “hard” blocks within the triblock copolymers. Once the macro-CTAs were synthesized and purified, their compositions were determined by ^1^H NMR spectroscopy and their molar masses were determined using size-exclusion chromatography (SEC, Table 1). It was found that the composition of all three of macro-CTAs was 42:58 (AA:St, Figure S1), which deviates slightly from the initial monomer feed ratio (50:50). The observed deviation is consistent with the reactivity ratios for this monomer pair (e.g., *r*_St_ = 0.15 and *r*_AA_ = 0.25, bulk polymerization),^59^ which should result in a slight enrichment in styrene content at lower total monomer conversions. From the reactivity ratios, it could be expected that at a targeted 50:50 (AA:St) composition a block with a close to alternating component sequence should form. However, since there is a lower mole fraction of AA in the final synthesized copolymer composition, styrene-rich regions are less likely. The DPs of the three macro-CTAs were determined by NMR spectroscopy to be 112, 216, and 280 (Table 1). However, these macro-CTAs were synthesized with a symmetrical bifunctional RAFT agent (Figure 1a), meaning that the trithiocarbonate functional group of the RAFT agent was located approximately in the middle of the macro-CTA (Figure 1a).^60^ Therefore, the copolymer will grow from the middle when chain extended to create an A_*x*_B_*y*_A_*x*_ triblock structure, where the A blocks will be half the length of the respective macro-CTA (i.e., *x* = DP/2 = 56, 108, and 140). SEC analysis showed that the molar masses of the macro-CTAs increased with the DP, and all the copolymers had relatively low dispersity (*M*_w_/*M*_n_ < 1.3, Table 1).

The macro-CTAs were chain-extended using an automated synthesizer that targeted six different DPs for the central poly(butyl acrylate) “B” block for each macro-CTA (Figure 1b, 18 triblock copolymer compositions in total). Since the *T*_g_ of PBA is about −50 °C,^61^ the BA blocks will be denoted as the “soft” blocks within the triblock copolymers. The BA block lengths were chosen such that the DPs of both the hard (A) and soft (B) blocks were varied as well as the total DP of the triblock copolymer (Figure S2). All the copolymerizations were performed at 40% w/w in MEK and reached moderate to high monomer conversions (70–95%) within 8 h (Table 2; specific reaction quantities can be found in Table S1). However, the conversions were lower when the longest macro-CTA (A_140_) was used, with these triblocks having an average conversion of 74%. SEC analysis of the triblock copolymers confirmed that a high blocking efficiency was achieved with BA and that *M*_n_ increased as the targeted DP of the soft block increased. In all cases, the triblock copolymers had dispersities less than or equal to 1.43 (Table 2 and Figure S3).

**Table 2:** Conversion and Molar Mass of the P(AA-*st*-St)-*b*-PBA-*b*-P(AA-*st*-St) Triblock Copolymers

		NMR			SEC^a^		
macro-CTA	triblock	targeted DP (BA)	conversion (%)	synthesized DP (BA)	*M*_n_ (kDa)	*M*_w_ (kDa)	*M*_w_/*M*_n_
A_56_A_56_	A_56_B_100_A_56_	100	89	89	11400	15400	1.34
	A_56_B_150_A_56_	150	93	140	13400	18100	1.35
	A_56_B_200_A_56_	200	92	184	14500	19700	1.25
	A_56_B_300_A_56_	300	92	276	18500	25000	1.35
	A_56_B_500_A_56_	500	92	460	24800	33100	1.34
	A_56_B_750_A_56_	750	88	660	32200	41500	1.29
A_108_A_108_	A_108_B_100_A_108_	100	78	78	17300	21600	1.25
	A_108_B_150_A_108_	150	82	123	18000	23000	1.28
	A_108_B_200_A_108_	200	86	172	21300	26200	1.23
	A_108_B_300_A_108_	300	85	255	19897	26300	1.32
	A_108_B_500_A_108_	500	88	440	25300	35000	1.38
	A_108_B_750_A_108_	750	86	645	29300	42000	1.43
A_140_A_140_	A_140_B_100_A_140_	100	74	74	20500	25800	1.26
	A_140_B_150_A_140_	150	72	108	22500	27900	1.24
	A_140_B_200_A_140_	200	71	142	22900	29100	1.27
	A_140_B_300_A_140_	300	76	228	24900	32300	1.30
	A_140_B_500_A_140_	500	72	360	25300	32200	1.28
	A_140_B_750_A_140_	750	75	563	34600	47200	1.37

a SEC measurements were performed using a THF eluent containing 1% v/v acetic acid against PSt standards on an APC instrument.

### Structure of the Macro-CTAs and Triblock Copolymers in MEK and Water Solutions

**Figure 2:**
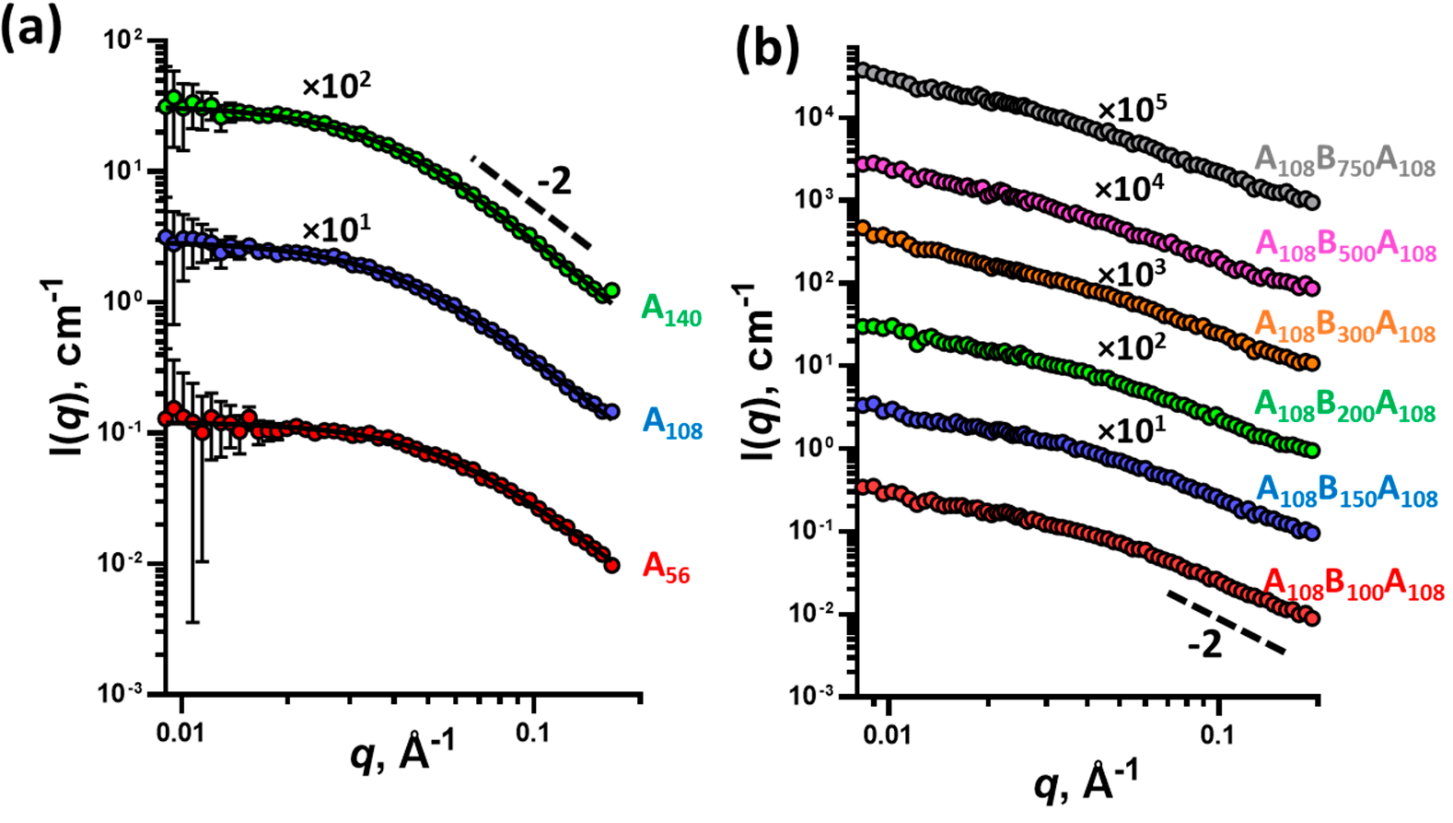
SAXS patterns of (a) 1% w/w macro-CTA solutions in MEK (symbols) fitted using the Debye function (eq S8) (black line) and (b) 1% w/w triblock copolymer solutions in MEK (symbols). Some patterns have been shifted upward (the multiplication factors are indicated on the plots) to avoid overlap. Scattering patterns were collected using a Bruker AXS Nanostar instrument.

The structural behavior of the synthesized copolymers was investigated in MEK, which is a reasonable solvent for polystyrene and poly(butyl acrylate), and water, where polystyrene and poly(butyl acrylate) are insoluble. Copolymers were first dissolved in MEK to form 1% w/w solutions and then analyzed using SAXS. SAXS patterns of the macro-CTAs (Figure 2a) show a clear plateau in scattering intensity in the low *q*-region (*q* < 0.04 Å^–1^) and a slope with a gradient close to −2. This scattering is indicative of a Gaussian coil. Consequently, these scattering patterns were all modeled using the Debye function (eq S8). Using this model, the radius of gyration (*R*_g_) of the copolymer chains was calculated; as expected, *R*_g_ increased monotonically as the length of the copolymer chain increased (*R*_g_ = 27, 37, and 42 Å for A_56_A_56_, A_108_A_108_, and A_140_A_140_, respectively).

The SAXS patterns collected for the triblock copolymers in the MEK solution (Figures 2b and S4) are characteristic of adjacent coils of copolymer blocks. Scattering intensity slopes close to −2 were observed at high *q*-values for the SAXS patterns, with no evidence of phase separation. This indicates that MEK is a reasonable solvent for the synthesized triblock copolymers. The SAXS analysis is also consistent with the visual observation of the triblock copolymer MEK solutions, which appear as clear transparent liquids. Nevertheless, it must be noted that a nonzero gradient at low *q*-values, which changes slightly with the copolymer composition, suggests that some of the chains interact with each other and possibly form larger objects. As a result, the radius of gyration of the copolymer blocks could not be obtained. However, it must be stressed that these interactions are weak and that the triblocks are in an unconstrained structure.

**Figure 3:**
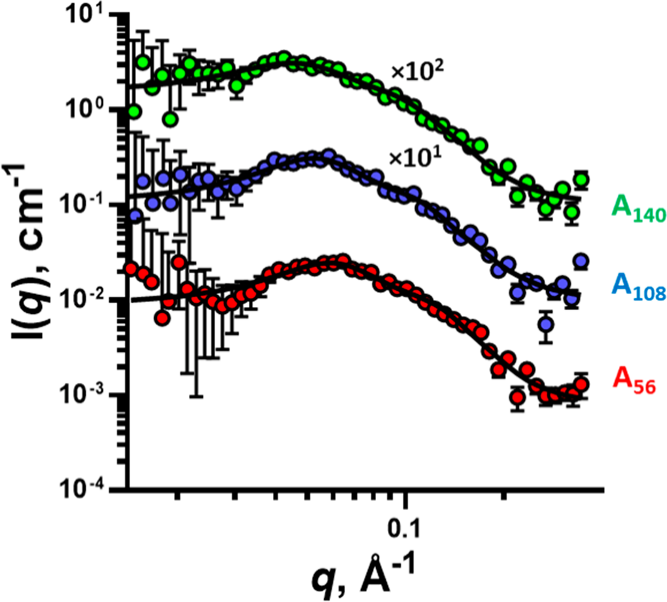
SAXS patterns of macro-CTA (A_56_A_56_, A_108_A_108_, and A_140_A_140_) aqueous dispersions (symbols) at a 1% w/w concentration. Patterns were fitted with the sphere model (eqs S9–S12) (solid line). Some patterns have been shifted upward (the multiplication factors are indicated on the plot) to avoid overlap. Scattering patterns were collected using a Bruker AXS Nanostar instrument.

The macro-CTAs (A_56_A_56_, A_108_A_108_, and A_140_A_140_) were formulated in water using a solvent-switch method in which the copolymers were initially dissolved at 75% w/w in IPA, then the mixtures were diluted with ammonia and water to produce stable dispersions. The macro-CTA dispersions were further diluted with water to 1% w/w for the SAXS analysis (Figure 3).

The SAXS patterns of the macro-CTA dispersions (Figure 3) show that these copolymers self-assemble into particulate structures, and the patterns can be fitted with a simple-sphere structural model (eqs S9–S12). However, a simple hard-sphere structure factor was also incorporated based on the Percus–Yevick approximation (eq S7) to account for the long-range charge interactions of the particles (Figure 5), as has been seen in similar systems.^51,62^ All macro-CTAs formed spherical particles of a similar size (*R* = 17 Å) independent of their molar masses. This is consistent with previous reports for statistical copolymers, where it was found the copolymer composition, rather than degree of polymerization, controlled the particle size.^42,51^ As the particle size remains constant while the molar mass of the copolymer increases, the aggregation number of the particle must decrease. Therefore, A_56_A_56_ particles will have a larger aggregation number than either A_108_A_108_ or A_140_A_140_ particles.

**Figure 4:**
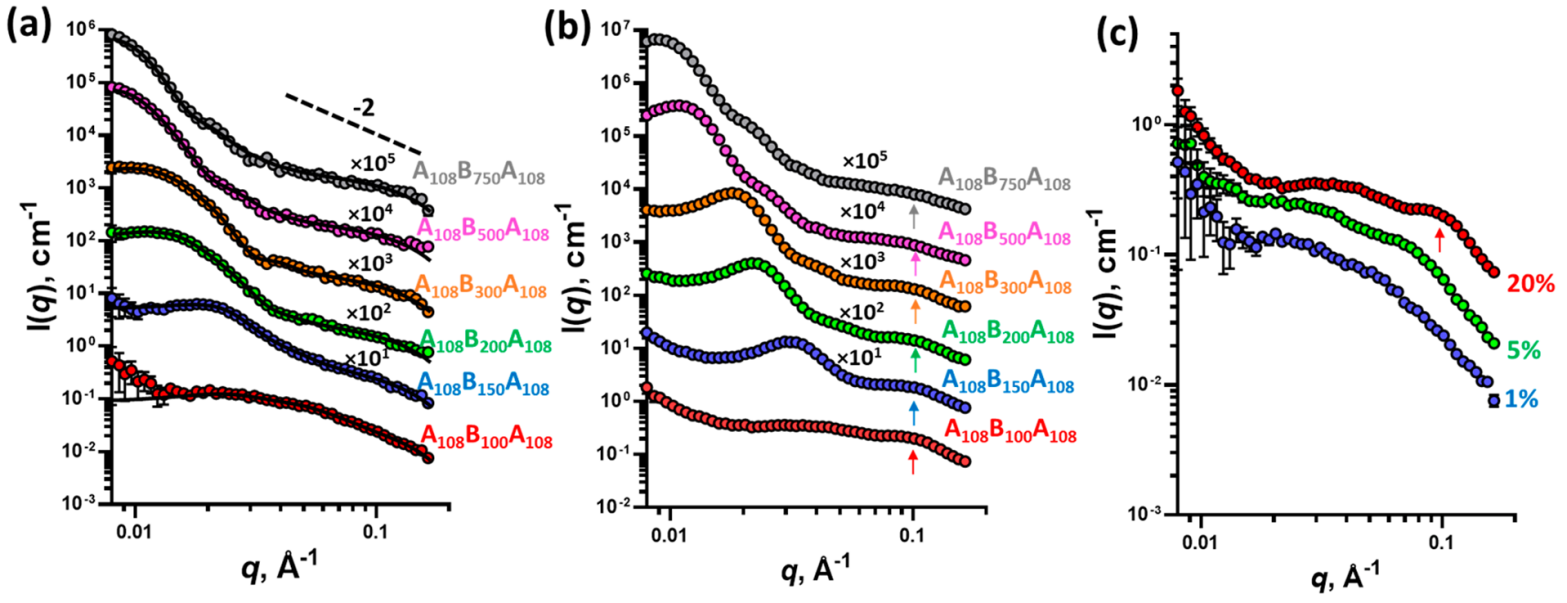
SAXS patterns of (a) 1% w/w A_108_B_100–750_A_108_ triblock copolymer aqueous dispersions (symbols) fitted with the two-population model (eqs S13–S15) (solid line); (b) A_108_B_100–750_A_108_ triblock copolymer aqueous dispersions at 20% w/w concentration; and (c) A_108_B_100_A_108_ triblock copolymer aqueous dispersion at 1, 5, and 20% w/w concentrations. The arrow is used to highlight the presence of the structural peak referred to in the text. Some patterns have been shifted upward (the multiplication factors are indicated on the plots) to avoid overlap. Scattering patterns were collected using a Bruker AXS Nanostar instrument.

Aqueous dispersions of the triblock copolymers were formulated using the same solvent-switch method used for the macro-CTAs and then diluted to 1% w/w in water for the SAXS analysis. It is clear from the SAXS patterns collected for the 1% w/w triblock copolymer dispersions (Figures 4a and S5) that these copolymers self-assemble to form particles, presumably through the hydrophobic interactions within the BA block, which induce copolymer aggregation to reduce any unfavorable interactions between BA and water. The positions of the form factor intensity minima move to lower *q*-values as the length of the BA block increases, suggesting that the particle size increases with BA DP (Figure 4a). Again, a broad peak in the scattering pattern, caused by the electrostatic particle–particle interactions, was observed in the Guinier region (low *q*-region) for most of the collected patterns. This peak feature can be fitted by incorporating the structure factor for hard spheres (eq S7) into the SAXS model, similar to the macro-CTA dispersions.

Typically, dispersions of spherical block copolymer particles produce scattering patterns with a −2 gradient of scattering intensity in the high *q*-region, as the soluble stabilizer block behaves like a Gaussian chain. However, gradients shallower than −2 were observed in the high *q*-regions (*q* > 0.08 Å^–1^) of the scattering patterns for the majority of triblock copolymer aqueous dispersions (Figure 4a). This suggests that there is an additional structural feature that produces scattering within the high *q*-region and that the source of the scattering has a relatively short length scale in comparison to the size of the primary copolymer particle. Additionally, this high-*q* feature is more prominent in the copolymers with greater weight fractions of the P(AA-*st*-St) stabilizer blocks (e.g., A_140_B_100_A_140_) (Figure 4b) and is clearly evident when the concentration of the dispersion is higher (indicated by the arrows in Figures 4b and c). This suggests that the high-*q* feature is related to an additional structural organization within the stabilizer block.

**Figure 5:**
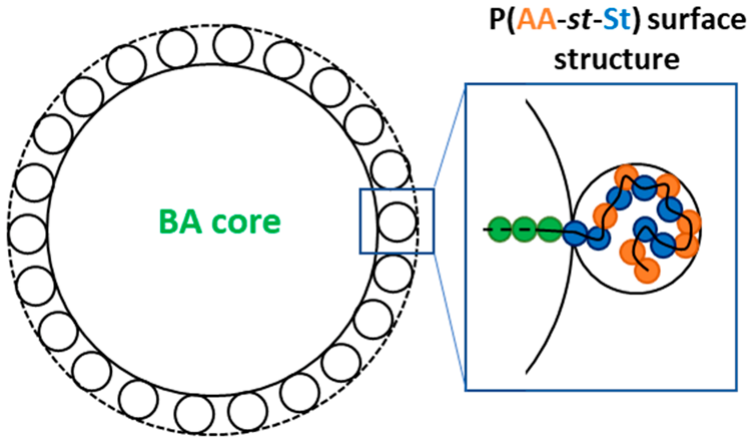
A diagram describing the predicted structure of a particle formed by P(AA-st-St)-*b*-PBA-*b*-P(AA-st-St) triblock copolymers in water, where the P(AA-st-St) stabilizer block was confined by the copolymer particle surface and “self-folded” through hydrophobic interactions.

The P(AA-*st*-St) block is expected to be located at or near the particle surface. Consequently, two potential explanations were considered: (1) there are SLD fluctuations within the corona block of the particle caused by the statistical distribution of styrene and acrylic acid and (2) the hydrophobic nature of styrene causes the statistical hard block to phase-separate within the particle surface through hydrophobic interactions in a manner similar to that observed for aqueous dispersions of amphiphilic statistical copolymers^42,51^ and in the SAXS analysis of the aqueous dispersions of the macro-CTAs.^47^ Both cases appear to be valid explanations for this phenomenon; however, the observation that the macro-CTAs will undergo spontaneous self-assembly to form spherical particles (Figure 3) indicates that the amphiphilic nature of the stabilizer block is enough to initiate the self-folding of the chain (Figure 5). The presence of this additional surface structure is caused by the hydrophobicity of the polystyrene units along the stabilizer backbone. This surface structure is not found in block copolymer dispersions with a fully hydrophilic stabilizer block [e.g., poly(acrylic acid)].^63−65^

Since the stabilizer block of the copolymer particle provides an additional structural morphology that is assumed to be spherical based on the behavior of the macro-CTAs in water (Figure 3) and thus does not behave as a Gaussian chain, the standard copolymer micelle structural model^66^ cannot be fitted to the SAXS patterns of the triblock copolymer dispersions. Instead, a two-population structural model that accounts for the spherical structures on the surface of the copolymer particle (Figure S6), as described in the Supporting Information (eqs S13–S15), was used to fit the SAXS data (Figure 4a). This model was developed to analyze polymer–silica colloidal particles,^67^ where the silica forms a particulate shell around a spherical polymer core.

For simplicity, the radius of the surface P(AA-*st*-St) spheres (*r*_2_) was fixed while fitting the SAXS patterns. It was assumed that these surface spheres were unimolecular (i.e., the statistical block formed a self-folded chain or a single-chain nanoparticle); therefore, the radius is given by , where *V*_AA-St_ is the volume of the statistical copolymer block and is directly related to the DP of the respective macro-CTA. The *V*_AA-St_ values for A_56_A_56_, A_108_A_108_, and A_140_A_140_ were calculated to be 6988, 13477, and 17470 Å^3^, respectively. Therefore, *r*_2_ values for the A_56_B_100–750_A_56_, A_108_B_100–750_A_108_, and A_140_B_100–750_A_140_ particles were fixed at 12, 15, and 16 Å, respectively. The core radius of the main particle, *R*_core_, was fitted during the SAXS model analysis (Tables 3 and S2).

**Table 3:** Structural Analysis Results for P(AA-*st*-St) and P(AA-*st*-St)-*b*-PBA-*b*-(AA-*st*-St) Copolymer 1% w/w Aqueous Dispersions^a^

copolymer	*R*_core_ (Å)	σ_core_ (Å)	*r*_2_ (Å)
A_56_A_56_	16^b^	5	
A_108_A_108_	16^b^	7	
A_140_A_140_	17^b^	7	
A_56_B_100_A_56_	53	14	12
A_56_B_150_A_56_	90	13	12
A_56_B_200_A_56_	119	18	12
A_56_B_300_A_56_	156	20	12
A_56_B_500_A_56_	247	28	12
A_56_B_750_A_56_	281	52	12
A_108_B_100_A_108_	18	9	15
A_108_B_150_A_108_	55	34	15
A_108_B_200_A_108_	99	24	15
A_108_B_300_A_108_	120	22	15
A_108_B_500_A_108_	191	39	15
A_108_B_750_A_108_	219	52	15
A_140_B_100_A_140_	15	5	16
A_140_B_150_A_140_	26	10	16
A_140_B_200_A_140_	32	23	16
A_140_B_300_A_140_	101	41	16
A_140_B_500_A_140_	150	41	16
A_140_B_750_A_140_	219	50	16

a *R*_core_ is the mean particle core radius, σ_core_ is the standard deviation of the mean particle core radius, and *r*_2_ is the radius of the self-folded P(AA-*st*-St) chain on the surface of the particle (this value based upon the volume of the hard block is fixed throughout the fitting).

b Note: *R*_core_ refers to the total particle radius for the A_56_A_56_, A_108_A_108_, and A_140_A_140_ macro-CTAs.

The modeling results show a clear trend in particle size, where the core radius increases as the length of the hydrophobic core-forming B block increases. Additionally, the core radius increases as the stabilizer block length decreases. These two influencing factors are commonly seen for block copolymer nanoparticle assembles.^8^ Furthermore, when the triblock copolymers possess very large stabilizer blocks (e.g., the A_140_B_100–750_A_140_ copolymers), their aggregation into larger particles appears to be significantly hindered until the B block DP reaches 300, with the core radii for A_140_B_100_A_140_, A_140_B_150_A_140_, and A_140_B_200_A_140_ remaining between 10 and 40 Å (Table 3). This behavior is seen at the beginning of the A_108_B_100–750_A_108_ series as well. The observed effect likely originates from a geometrical confinement caused by the copolymer aggregation number and the packing density of the A block spheres on the triblock copolymer particle surface (Figure 5). The relatively large spheres formed by the A blocks are comparable with the *R*_core_ of the triblock copolymer particle core formed by the relatively short B block, restricting the formation of larger particles. When the B block becomes significantly larger than the A block, this effect is negligible.

These results demonstrate that copolymer particles can be successfully stabilized as a dispersion in water using an amphiphilic statistical stabilizer block rather than a wholly hydrophilic block. Additionally, particles display an interesting surface structure due to the hydrophobic units within the stabilizer block. The hydrophobic units cause the chains to collapse into single-chain assemblies on the particle surface. These hydrophobic surface domains are similar to the structures observed for the orthogonal folding of random block copolymers.^49^ Finally, these results demonstrate that the particle size can be easily tuned by varying the DP of either the core block or the amphiphilic stabilizer block, as seen in other triblock copolymer systems.

### Structural Characterization of Triblock Copolymer Films Cast from an Organic Solvent

The analysis of the triblock copolymers in both MEK and aqueous media demonstrate that the copolymers form very different morphologies depending on their interactions with the solvents. In MEK, a reasonably good solvent for all the copolymer components, the copolymers were found to be dissolved chains with some weak association into loose aggregates, whereas in water the copolymers assembled into well-defined spherical particles with a particulate shell. Such a different solution behaviors of the synthesized copolymers could be reflected in the bulk morphologies of films cast from the triblock copolymer solutions or dispersions. Therefore, triblock copolymer films were prepared from both the organic solutions and the aqueous dispersions. The presence and type of structural phase separation within these films were assessed by SAXS and atomic force microscopy (AFM) (see the Supporting Information).

**Figure 6:**
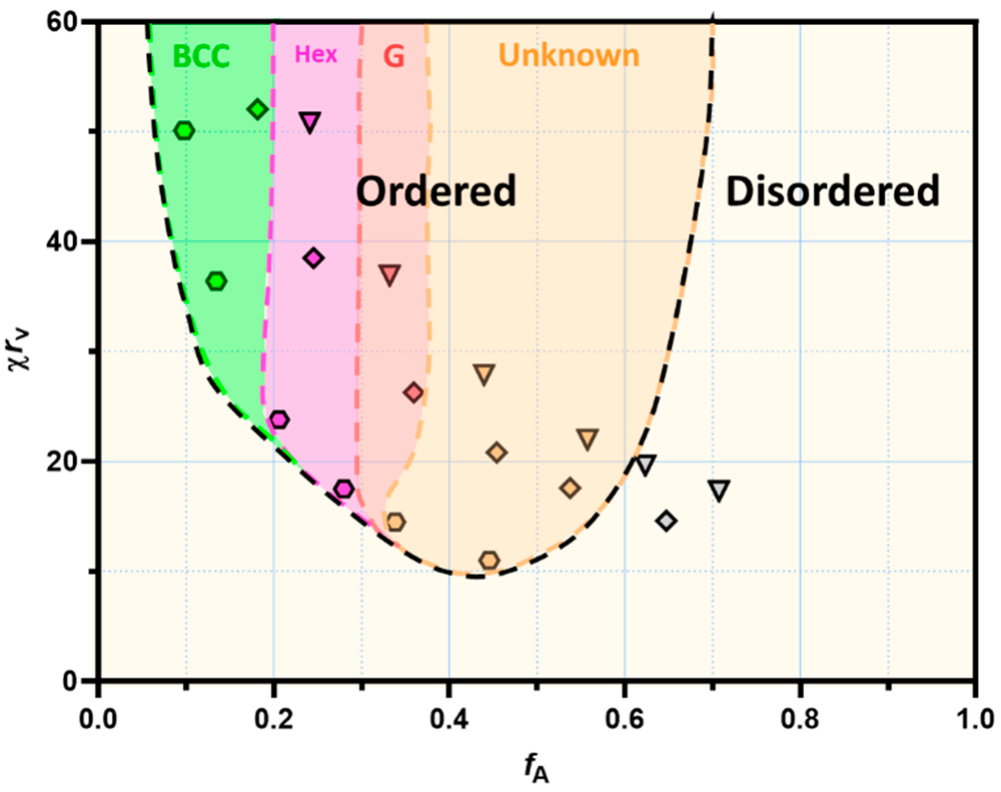
Predicted phase diagram of the bulk triblock copolymer morphologies based on SCFT theory, AFM images, and SAXS, where *f*_A_ is the volume fraction of the A block, χ is the Flory–Huggins segment interaction parameter, and *r*_*v*_ is the overall length of the copolymer molecule in segments, taking into account the difference in the volume occupied by the A and B block. *r*_v_ was calculated using the equation *r*_v_ = *x* + *y*(*v*_B_/*v*_A_) where *v*_A_ and *v*_B_ are the averaged volumes occupied by the A and B monomer units, respectively.^69^ The A_56_B_*y*_A_56_, A_108_B_*y*_A_108_, and A_140_B_*y*_A_140_ series are represented by hexagon, diamond, and triangle symbols, respectively. BCC, Hex and G indicate zones of existance of body-centered cubic, hexagonally-packed cylinder and gyroid copolymer phases, respectively.

The volume fraction of the A (hard) blocks (*f*_A_) in the composition of the synthesized triblock copolymers changes for the A_56_, A_108_ and A_140_ series from 0.13 to 0.45, 0.18 to 0.65, and 0.24 to 0.71, respectively. It has to be pointed out that the A_108_B_100_A_108_, A_140_B_100_A_140_, and A_140_B_150_A_140_ films, which are associated with largest volume fraction of hard A block (*f*_A_) in their copolymer series, show no structural peaks indicating phase separation (Figure S6). On the basis of a phase diagram predicted by self-consistent field theory (SCFT) for symmetrical triblock copolymers,^68^ this observation suggests that the A_108_B_100_A_108_, A_140_B_100_A_140_, and A_140_B_150_A_140_ copolymers form disordered structural morphologies (Figure 6). In contrast, the A_56_B_100_A_56_ film, with the largest *f*_A_ value of A_56_ series, shows a structural peak indicating an ordered structure (Figure S6a). Thus, the A_56_B_100_A_56_ copolymer with *f*_A_ close to 0.5, which is usually associated with the critical point of copolymer phase diagrams, is likely to be in the ordered structural morphology region of the phase diagram (Figure 6).

According to a theoretical symmetrical triblock copolymer phase diagram predicted by SCFT, the triblock copolymer morphologies in the bulk change as a factor of the block volume fraction *f*.^68^ In particular, SCFT suggests that as *f*_A_ decreases (i.e., 0.45 > 0.36 > 0.25 > 0.18) the morphology will transition from lamellae, to a gyroid, then to hexagonally packed cylinders, and finally to a body-centered cubic structure.^70^ The sequence of phases observed by AFM in this work for triblock copolymers with statistical side blocks (Figures S8 and S9) agrees reasonably well with the symmetrical triblock copolymer phase diagram. For example, the A_108_ series shows transitions from a bicontinuous structure (likely to be gyroid) to cylinders to spheres as the BA block increases in size (i.e., as *f*_A_ decreases). Furthermore, a phase diagram of the bulk triblock copolymer morphologies predicted with respect to χ*r*_v_ (the product of the Flory–Huggins interaction parameter, χ, and the overall copolymer length in the segments, *r*_v_(69)) and *f*_A_ was constructed using SCFT theory, AFM images and SAXS data (Figure 6). It was assumed that the A_56_B_100_A_56_ copolymer composition with a *f*_A_ value about 0.5 was close to the composition corresponding to the critical point of the copolymer phase diagrams, with χ*r*_v_ = 10.5. Since the A_56_B_100_A_56_ copolymer shows phase separation and should belong to the ordered region of the phase diagram, its χ*r*_v_ value was taken to be 11.0, which was slightly above the order–disorder boundary (Figure 6). The combined analysis of AFM images (Figure S8) and SAXS results (Figure S6), summarized in Table S3, enabled approximate boundaries between ordered phases to be established for the soft (B) block-rich compositions (Figure 6, left side of the diagram). Some of the observed morphologies appear to have some mixed phases (e.g., the A_108_B_750_A_108_ image shows small cylinders and spheres, Figure S8). The lack of order could be caused by both nonequilibrium conditions of the film preparations and relatively large dispersity in the chain lengths (Table 2).^71^ While the order–disorder transition for the hard-block-rich compositions was evident from the SAXS measurements (Figure S6), it was not possible to identify the ordered phase structures (Figure 6, left side of the diagram). It could be expected that the statistical nature of the hard blocks would affect the ordered phase symmetry, resulting in distorted (ill-defined) structures. Thus, contrasting behavior between block copolymers and statistical copolymers may cause the triblock copolymer phase diagram to be asymmetrical, with the homogeneous (B) block-dominating side represented by well-ordered phases expected for block copolymers and the statistical (A) block-dominating side represented by ill-defined structures (Figure 6). The obtained results demonstrate that varying the DP of the either the hard block or the soft block has a direct effect on both object size and interobject distances and that the copolymer composition determines the phase-separated morphology formed by self-assembled P(AA-*st*-St)-*b*-PBA-*b*-P(AA-*st*-St) macromolecules in the film when cast from MEK.

### Structural Characterization of Triblock Copolymer Films Cast from Aqueous Dispersions

**Figure 7:**
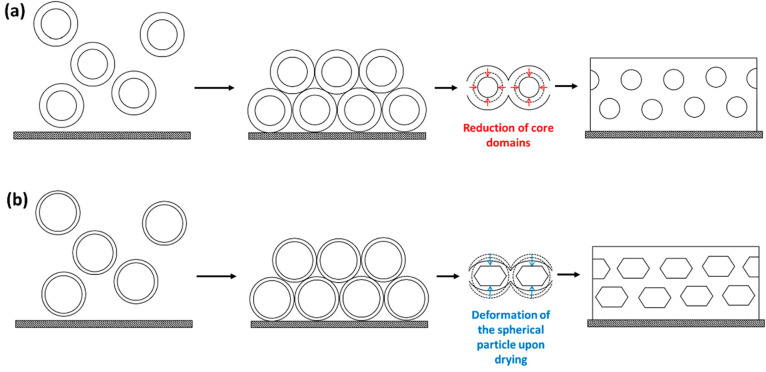
Schematics of the formation of the kinetically trapped structure within the water-cast films that occurs during the drying process. (a) The formation of the packed soft sphere structure within a matrix of the hard phase, where the BA-reach domains reduce during the drying process. (b) The formation of a structure of distorted packed spheres that is formed by copolymers with a large soft block.

To assess how the solvent environment affected the phase-separated structure in the film, films were also cast from aqueous dispersions of the copolymers and analyzed by SAXS and AFM as before (see the Supporting Information). A primary scattering intensity peak observed in the SAXS patterns of this triblock films demonstrates (Figure S13) that phase separation is present in all the films. This was not the case for the films cast from MEK, where A_108_B_100_A_108_, A_140_B_100_A_140_, and A_140_B_150_A_140_ triblock copolymers had little or no phase separation (Figure S6). This comparison suggests that film-casting from an aqueous environment facilitates phase separation, presumably because the initial aqueous dispersions are already phase-separated into particles to minimize the strongly unfavorable interactions between the PBA block and water. Therefore, when an aqueous film is cast, the copolymers are largely preassembled with aggregated regions of hard and soft blocks, making it easy for large-scale phase separation to occur within the film (Figure 7). Conversely, in MEK, the triblock copolymers are molecularly dissolved and therefore not phase-separated in the solution, making it more difficult to induce phase separation within the film. This can be thought of in terms of Flory–Huggins interaction parameters. Since the copolymers are the same in each case, χ will be the same between the blocks. However, the polymer–solvent interaction parameter will be different based on whether the copolymer is in water or MEK. In the case of the PBA (soft) block, χ will be high for water but lower for MEK, since PBA is insoluble in water but soluble in MEK. The fact that the A_108_B_100_A_108_ films show little phase separation when cast from MEK suggests that a higher DP is required for the given χ of the blocks to induce phase separation. However, the high χ between the PBA block and water drives the self-assembly in water, which remains within the water-cast films.

Previous studies investigating the structures of similar diblock copolymer spherical micelle films suggest that while the spherical cores keep their shape as the film dries, the corona blocks coalesce to form a continuous matrix (Figure 7).^27^ This phenomenon will produce different scattering patterns depending on how the particles stack upon drying. A well-ordered cubic structure, such as body-centered cubic and face-centered cubic structures, would produce sharp diffraction peaks related to the crystallographic planes of the crystal structure. However, the water-cast films of P(AA-*st*-St)-*b*-PBA-*b*-P(AA-*st*-St) show relatively broad peaks (Figure S13) similar to the scattering patterns observed for randomly packed spherical particles.^72−74^ Indeed, the SAXS patterns of the majority of the water-cast triblock copolymer films (A_56_B_100–300_A_56_, A_108_B_100–300_A_108_, and A_140_B_100–300_A_140_) were fit using a spherical form factor (eqs S9–S12) combined with a structure factor of interacting hard spheres (eq S7) (Figure S15). This result supports the observations made in other studies on similar systems.^26,27,33,75,76^ However, the additional features at high *q*-values, which are associated with the packing of particles formed by the hard (A) block and the packing of the BA chains (for further discussion, see the Supporting Information), are not accounted for within this structural model and are not fitted by the model. The results from this modeling indicate that the spherical domain size increases as the length of the hydrophobic BA block increases; this observation is similar to the observation for the triblock copolymer dispersions (Table S4).

The triblock films that do not fit to the spherical particle model are the copolymers that have large volume fractions of the PBA component, i.e., A_56_B_500_A_56_, A_56_B_750_A_56_, A_108_B_500_A_108_, A_108_B_750_A_108_, A_140_B_500_A_140_, and A_140_B_750_A_140_. This suggests that the particles within these copolymer films arrange in a different structure compared to the previously examined films. Assuming that the particle PBA cores form a close-packed structure of spheres upon drying and that the residual volume is filled by the hard P(AA-*st*-St) matrix, a maximum volume fraction of about 0.74 can be filled by the undistorted spherical PBA cores. However, all the copolymer films produced SAXS patterns that did not follow the spherical form factor model (eqs S7 and S9–S12) and had soft PBA block volume fractions greater than 0.74. This means that the spherical cores will distort during the drying process in order to reduce the residual volume (Figure 7b); therefore, this structure cannot be analyzed by the sphere model.^77,78^

AFM images of the water-cast films (Figure S16), unlike the AFM images of the solvent-cast films (Figure S8), show that there is little surface phase separation, and there is no long-range structural order visible in the images of the water-cast films. This is consistent with the SAXS results that suggest a randomly packed (amorphous) structure formed by spheres. The absence of well-defined phase separation is likely due to the lack of mobility that would allow the blocks to rearrange upon drying, with the majority of the soft hydrophobic blocks potentially buried within a continuous surface of the hard P(AA-*st*-St) matrix (Figure 7). Some films show surface structure that is thought to be caused by drying defects resulting in an uneven film (e.g., A_56_B_500_A_56_). However, images of the A_108_B_200_A_108_ and A_108_B_300_A_108_ films show a better-defined structural morphology, where the dark and light regions could correspond to spherical soft regions in a matrix of the hard phase (Figure S16).

### Structural Characterization of Annealed Triblock Copolymer Films Cast from Aqueous Dispersion

The results gathered through SAXS and AFM demonstrate that the solvent environment from which the copolymer film is cast plays a major role in the resultant structure within the film. The film structure is linked to the initial state of copolymer within the solution phase. If the copolymer is dissolved within the solvent phase (the MEK solvent case), then it will have the freedom to arrange into a more favorable and lower energy structure. However, if the copolymers are preassembled in solution through solvophobic interactions (the aqueous case), then their mobility is restricted and they are unable to rearrange into a favorable conformation upon drying.

**Figure 8:**
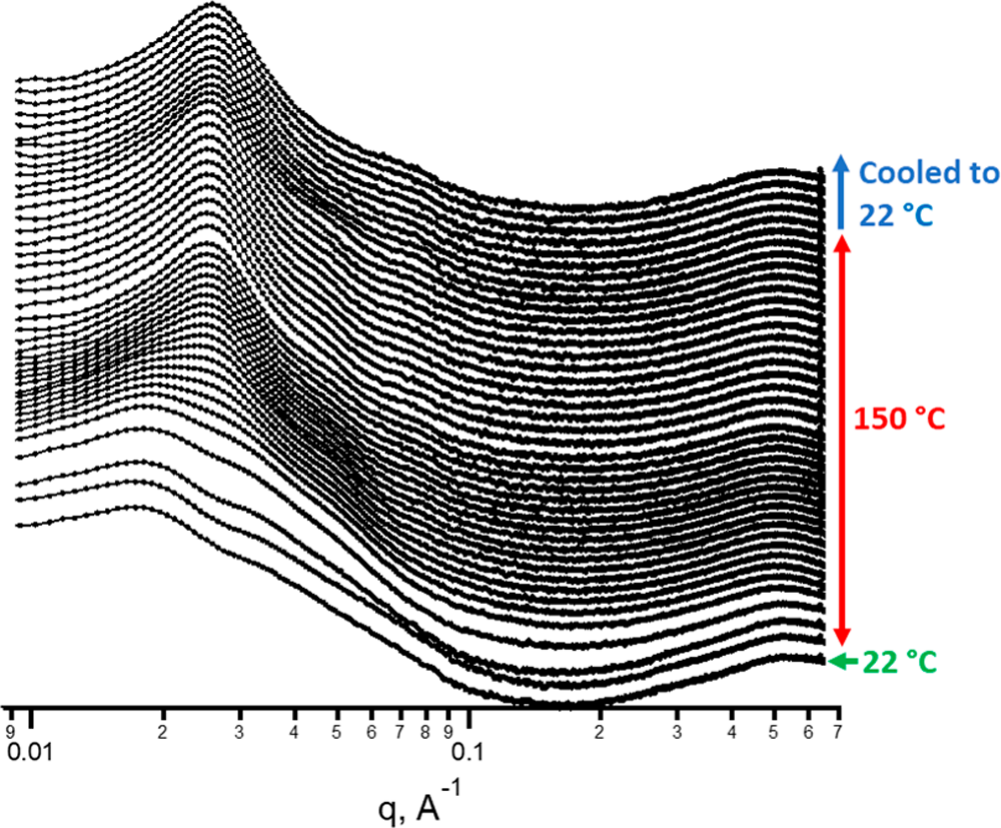
Time-resolved GISAXS patterns collected for the A_108_B_500_A_108_ water-cast film during the annealing process at 150 °C. The temperature protocol is shown at the right side of the plot. Scattering patterns were collected using a Xenocs Xeuss laboratory beamline.

The aqueous-cast films were thought to be in a kinetically trapped state due to the high *T*_g_ of the stabilizer block preventing the coalescence of the soft particle cores. Therefore, if the temperature is raised above the *T*_g_ of the hard block (∼120 °C), then the copolymer mobility should increase, allowing the structure to rearrange into a more thermodynamically stable conformation. To test this hypothesis, in situ grazing incident SAXS was performed on an aqueous-cast A_108_B_500_A_108_ film while the film was annealed (Figure 8). In this experiment, an initial scattering pattern was acquired at ambient temperature (22 °C). Afterward, the triblock film was heated to 150 °C, above the *T*_g_ of the hard block (120 °C), then frames were collected every 60 s to monitor any change in structure during the annealing process. The SAXS analysis shows that the structure present in the water-cast film at 22 °C begins to rearrange when heated to 150 °C. An equilibrated structure was eventually reached after 30 min, and the film was cooled to ambient temperature.

**Figure 9:**
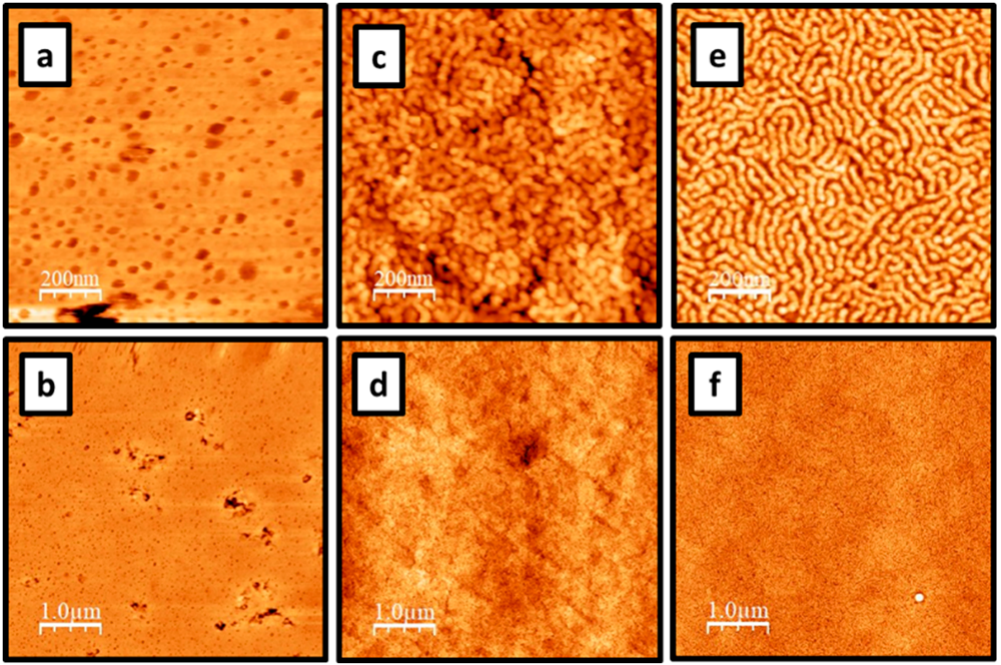
AFM height (tomographic) images of triblock copolymer films recorded using the ScanAsyst PeakForce tapping mode. (a and b) Images of A_108_B_500_A_108_ cast from a 20% w/w aqueous solution; images were recorded for 1 μm × 1 and 5 μm × 5 μm areas, respectively. (c and d) Images of A_108_B_500_A_108_ cast from a 20% w/w aqueous solution and annealed for 30 min at 150 °C; images were recorded for 1 μm × 1 and 5 μm × 5 μm areas, respectively. (e and f) Images of A_108_B_500_A_108_ cast from a 40% w/w solution in MEK; images were recorded for 1 μm × 1 and 5 μm × 5 μm areas, respectively.

The final SAXS pattern of the water-cast film taken after the annealing process is almost identical to the SAXS pattern collected for the solvent-cast film of the same triblock copolymer (Figures S13 and S14). Since a change was observed in the structure of the water-cast film once the temperature was raised above the *T*_g_ of both blocks, it is evident that the original structure was in a kinetically trapped state and a more thermodynamically stable structure could only be achieved by annealing. This rearrangement of the bulk structure, where the kinetically trapped hard matrix undergoes inversion when the film is annealed to form a structure wherein the hard domains are surrounded by a continuous matrix of the soft component, was reported previously.^27^ AFM images were taken of the film before (Figure 9a and b) and after (Figure 9c and d) annealing and compared with the images taken of the films cast from solvent in ambient conditions (Figure 9e and f). These images show a clear change in the phase-separated structure once the water-cast film is annealed. The AFM images also demonstrate that the structure of the annealed film is similar to that of the film cast from solvent in ambient conditions (Figure S8).

The SAXS and AFM data are in good agreement with each other. They demonstrate the structural differences in the films cast from different solvent environments and how the copolymer properties affect the phase separation within the films. When the triblock copolymer is fully dissolved in the solution phase, the triblock copolymer has a large amount of mobility that is not restricted by unfavorable interactions with the solvent. This mobility allows the copolymer to arrange into a lower-energy structure as the solvent evaporates and a film forms. However, when the selected solvent is not compatible with one of the copolymer blocks, then the triblock copolymers aggregate together to form particles to avoid any unfavorable solvent interactions. As a result, the copolymers have a restricted mobility and cannot rearrange into a thermodynamically stable structure, as the solvent evaporates and so remain kinetically trapped. Annealing the aqueous film above the *T*_g_ of the hard block provides mobility to the copolymer chains, allowing them to rearrange into a lower-energy structure. However, annealing the film does not provide as much mobility to the copolymer chains as their solvation by MEK, which is why the annealed film appears less well-defined in AFM images (Figure 9) and may require a longer annealing time to complete the morphology transformation.

### Mechanical Characterization of the Triblock Copolymer Films

The structural morphology is likely to have a significant impact on the mechanical properties of films. In order to establish structure–property relationships, the mechanical properties of water-cast films and solvent-cast films were investigated.

**Figure 10:**
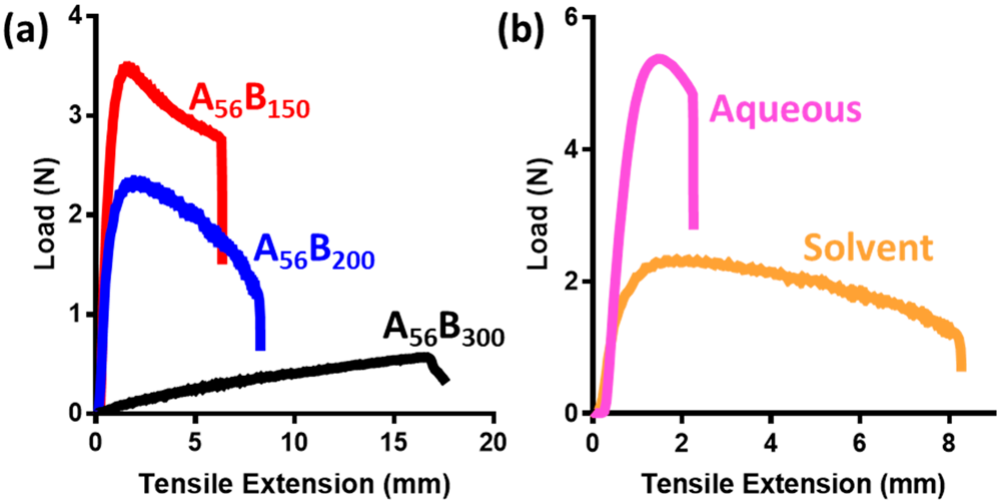
Tensile extension traces of triblock copolymer films. (a) Trace demonstrating the effect of increasing the length of the soft block when cast from MEK. (b) Trace demonstrating the effect of changing the solvent environment from which the A_56_B_200_A_56_ copolymer film is cast.

Since the lengths of the soft and hard blocks varied across all 18 triblock copolymers (Figure S2), a range of structural morphologies were achieved. However, some films were either too brittle or too soft to undergo mechanical testing. For the solvent-cast films, the mechanical tests show that increasing the length of the soft block systematically increases the flexibility of the film (higher extension-to-break and lower modulus, Table S5 and Figure 10a), while increasing the length of the hard block increases the film strength (higher modulus, lower extension-to-break, Table S5). This behavior is predictable, since it is well-known that, at a fixed temperature, polymers become more pliable by lowering their *T*_g_s.^79^ Therefore, by introducing a larger soft component to the copolymer, the triblock copolymer film has a larger volume of low-*T*_g_ phase. Furthermore, the hard segments within the triblock copolymer film will aggregate together (as indicated by SAXS and AFM) to form glassy regions that act as cross-linking points across the film. Therefore, increasing the length of the hard block increases the size of the glassy regions and consequently increases the strength of the cross-linking and the toughness of the triblock film.

Although the film properties are largely dictated by the copolymer composition and design, the solvent environment also has a significant effect on the film behavior. The mechanical tests indicate that a triblock copolymer film cast from water produces a harder film (higher modulus) than the same copolymer cast from MEK (Table S5 and Figure 10b). This is likely a result of the phase-separated structure within the film and the respective location of the hard and soft blocks. Casting the copolymer from water produces a kinetically trapped phase-separated structure in which the hard P(AA-*st*-St) block forms a continuous matrix. However, when conditions allow the copolymers to reach thermodynamic phase-separation the continuous phase is often formed by the soft block, as it is the larger component by volume. The variations of the initial copolymer state (either in a solution or in a dispersion) result in the different mechanical properties of dried films, which clearly demonstrates the importance of the solvent environment.

## Conclusions

In this study, a combination of statistical and triblock copolymer properties was explored with regard to their ability to stabilize hydrophobic particles and the properties of the polymers once cast into films. It is demonstrated that amphiphilic statistical A blocks of ABA triblock copolymers can provide sufficient stabilization to largely hydrophobic particles formed upon the self-assembly of the copolymers. This may allow the hydrophobicity of the stabilizer block to be tuned by varying the block composition, which may ultimately affect the copolymer morphology in solutions and solid films.

A bifunctional symmetrical RAFT agent was used for the RAFT synthesis of symmetrical ABA triblock copolymers with statistical copolymer A blocks. *S*,*S*-Dibenzyl trithiocarbonate was used in order to achieve symmetrical poly(acrylic acid-*stat*-styrene)-block-poly(butyl acrylate)-block-poly(acrylic acid-stat-styrene) triblock copolymers in two steps: (i) the synthesis of a poly(acrylic acid-*stat*-styrene) macro-CTA with a midchain trithiocarbonate and (ii) chain extension from the chain center with butyl acrylate to yield the desired triblock copolymer. Molar mass analysis demonstrated that the macro-CTAs were extended with high blocking efficiency and all had a relatively low dispersities (*M*_w_/*M*_n_ < 1.5).

SAXS analysis showed that the solubility of the triblock copolymer in MEK varied with the copolymer composition. However, none of the copolymers dissolved in MEK assembled into particles. In water, the triblock copolymers self-assembled into spherical particles. As expected, the particle size increased with the length of the hydrophobic soft block. It was found that the copolymer systems underwent a twofold self-assembly process when dispersed in water: (i) self-assembly of the hydrophobic core and (ii) self-assembly within the amphiphilic P(AA-*st*-St) stabilizer blocks, which form small, folded structures on the particle surface to reduce any unfavorable interactions with water.

SAXS and AFM analyses of organic solvent (MEK) cast films demonstrated that the length scale of the phase separation increased as the length of the soft [poly(butyl acrylate)] block increased. Additionally, the length scale of the phase separation increased as both the hard block length and the overall triblock length increased. The observed morphological transitions agreed well with the well-established phase separation of the diblock and symmetrical triblock copolymers, where the structural morphology changed from a gyroid, to cylinders, to spheres as the volume fraction of the soft block increases. It is also found that the packing of poly(butyl acrylate) blocks in the phase-separated copolymer structure is controlled by their relatively long pendent groups, which keep the copolymer backbones at a fixed distance from each other. AFM showed that the surface structure of the solvent cast films was highly dependent on the ratio of the hard and soft components. When there were significantly higher amounts of the soft block in the copolymer, the soft component would form the continuous phase and vice versa.

It is found that, analogous to the solvent-cast films, the copolymer phase-separation length scale of the aqueous-cast films increases with the lengths of the individual blocks. However, the phase-separation structure within copolymer films dried from aqueous dispersions is dominated by a close-packed sphere-like structure. This structure is formed by copolymer particles kinetically trapped due to the restricted movement of the blocks in the initial aqueous dispersion.

The mechanical properties of the solvent-cast copolymer films were consistent with the copolymer composition; the higher the hard block content in the films, the higher the elastic modulus and the lower the extension-to-break. In contrast, when the amount of the soft block increases, the elastic modulus decreases and the extension-to-break increases. However, the films cast from water did not show such an obvious trend, and the triblock copolymer films of different compositions show similar behavior. These similarities in the mechanical properties of the films are likely due to the similar structural morphologies formed from water by the triblock copolymers upon drying. The aqueous-cast copolymer films are characterized by a higher elastic modulus and a lower extension-to-break than their solvent-cast counterparts. This behavior is associated with the differences in the structural morphologies formed in these two systems, where the continuous phase of hard blocks, always formed in the case of the aqueous-cast films, produces a harder film. These results have demonstrated that the solution behavior of block copolymers has a direct effect on the film structure and the resulting mechanical properties.

## Supporting Information Available

Further characterization of triblock copolymer solutions, dispersions films, and SAXS models (PDF)
