# Supplementary material for: Solution and Solid-State Behavior of Amphiphilic ABA Triblock Copolymers of Poly(acrylic acid-stat-styrene)-block-poly(butyl acrylate)-block-poly(acrylic acid-stat-styrene)
Source: Macromolecules. 2022 Oct 28;55(21):9726–39. doi: 10.1021/acs.macromol.2c01299 (PMC9648343; doi:10.1021/acs.macromol.2c01299)
Supplement: Supplementary file 1 — ma2c01299_si_001.pdf [file ma2c01299_si_001.pdf]

Supporting Information:

Solution and solid state behavior of amphiphilic ABA triblock copolymers of poly(acrylic acid-*stat*-styrene)-*block*-poly(butyl acrylate)-*block*-poly(acrylic acid-*stat*-styrene)

Thomas J. Neal,[a] Robert D. Bradley,[b] Martin W. Murray,[b] Neal S. J. Williams,[b] Simon N. Emmett,[b] Anthony J. Ryan,[a] Sebastian G. Spain,[a,] Oleksandr O. Mykhaylyk.[a,*]*

[a] Department of Chemistry, Dainton Building, The University of Sheffield, Sheffield, S3 7HF, UK.

[b] AkzoNobel Decorative Paints, Wexham Road, Slough, Berkshire, SL2 5DS, UK

Contents

Characterisation of the synthesised triblock copolymers and their structural morphology in solutions/dispersions 3

Characterisation of the triblock copolymer films 8

Structural characterization of triblock copolymer films cast from an organic solvent 8

Diffraction peak indexing of SAXS patterns of dried P(AA-*st*-St)-*b*-PBA-*b*-P(AA-*st*-St) films cast from an organic solvent 17

Structural characterization of triblock copolymer films cast from aqueous dispersions 21

Mechanical characterization of the triblock copolymer films 27

SAXS Structural Models 28

Intensity equation 28

Intensity equation with linear background 28

Distribution function of the structural model parameters 29

Hard sphere structure factor 29

Form factor for a Gaussian chain 29

Sphere model 30

Two-population model 31

Gaussian distribution 32

References 33

Characterisation of the synthesised triblock copolymers and their structural morphology in solutions/dispersions

Table S1. Summary of reagent quantities required for the synthesis of the P(AA-*st*-St)-*b*-PBA-*b*-P(AA-*st*-St) triblock copolymers.

Triblock	Macro-CTA (g)	BA (g)	AIBN (g)[a]	MEK (g)[b]
$A_{56}B_{100}A_{56}$	4.36	5.50	0.0141	18.3
$A_{56}B_{150}A_{56}$	3.43	6.50	0.0110	18.5
$A_{56}B_{200}A_{56}$	2.97	7.50	0.0096	19.5
$A_{56}B_{300}A_{56}$	2.25	8.50	0.0070	20.0
$A_{56}B_{500}A_{56}$	1.43	9.00	0.0046	19.4
$A_{56}B_{750}A_{56}$	1.00	9.50	0.0033	19.5
$A_{108}B_{100}A_{108}$	7.54	5.00	0.0130	23.3
$A_{108}B_{150}A_{108}$	6.03	6.00	0.0100	22.4
$A_{108}B_{200}A_{108}$	5.28	7.00	0.0090	22.8
$A_{108}B_{300}A_{108}$	4.02	8.00	0.0070	22.3
$A_{108}B_{500}A_{108}$	2.71	9.00	0.0050	21.8
$A_{108}B_{750}A_{108}$	2.01	10.00	0.0030	22.3
$A_{140}B_{100}A_{140}$	8.76	4.50	0.0120	24.7
$A_{140}B_{150}A_{140}$	7.14	5.50	0.0090	23.5
$A_{140}B_{200}A_{140}$	6.33	6.50	0.0080	23.8
$A_{140}B_{300}A_{140}$	4.87	7.50	0.0060	23.0
$A_{140}B_{500}A_{140}$	3.51	9.00	0.0050	23.2
$A_{140}B_{750}A_{140}$	2.60	10.00	0.0030	23.4

[a] AIBN was added as a 14 mg mL^{-1} solution, the quantity presented in the table is the quantity of pure AIBN added to the reaction solution rather than the mass of the AIBN solution added. [b] Quantity of MEK presented here is the total quantity of MEK in the final reaction solution (i.e., MEK added combined with MEK with AIBN solution).

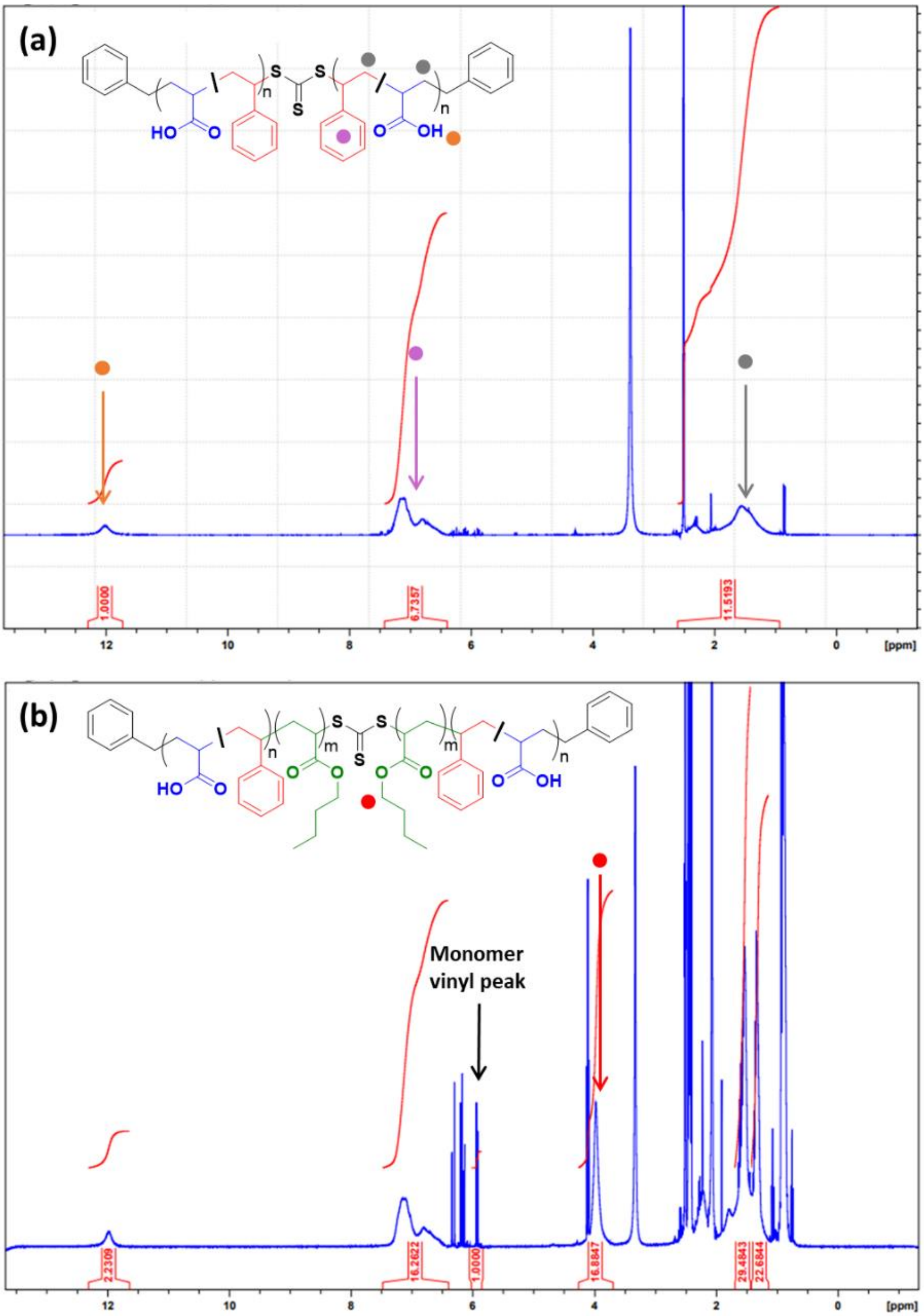

Figure S1. Example ^{1}H NMR spectra in deuterated DMSO of (a) the $A_{56}A_{56}$ macro-CTA, where the comparison of the styrene and acrylic acid integrals gives a copolymer composition of 42:58 (AA:St), and (b) the $A_{56}B_{150}A_{56}$ triblock copolymer, where the conversion was calculated by the comparison of the monomer and polymer integrals and was found to be 93%.

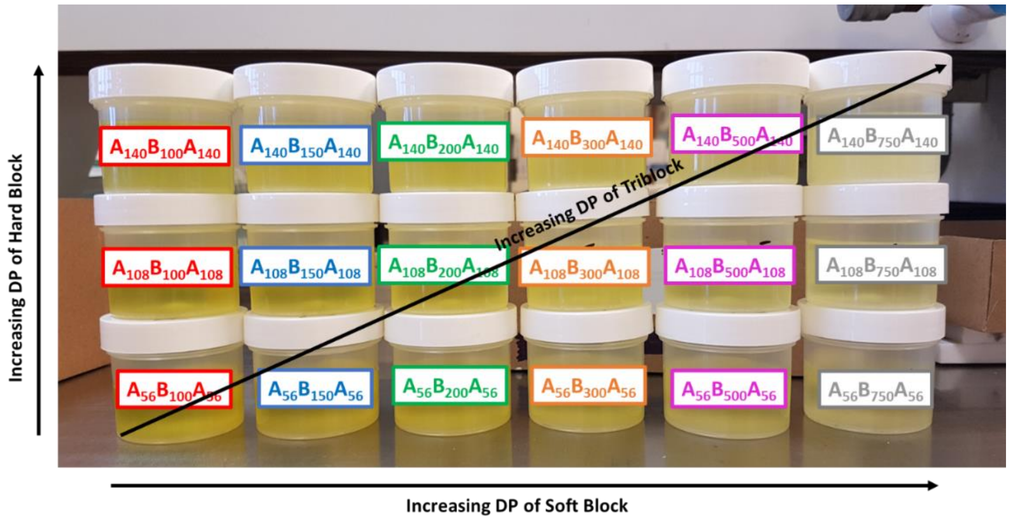

Figure S2. A photograph of the triblock copolymer solutions synthesised on the ChemSpeed High-Throughput Robot in MEK, depicting how the targeted copolymer composition is varied across the triblock copolymer series.

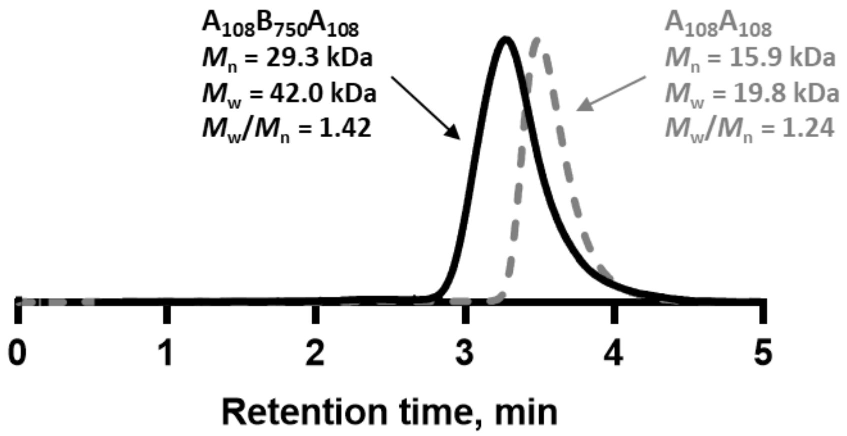

Figure S3. An example APC trace of $A_{108}B_{750}A_{108}$ triblock copolymer (black solid line) and $A_{108}A_{108}$ macro-CTA (grey dashed line) demonstrating the blocking efficiency.

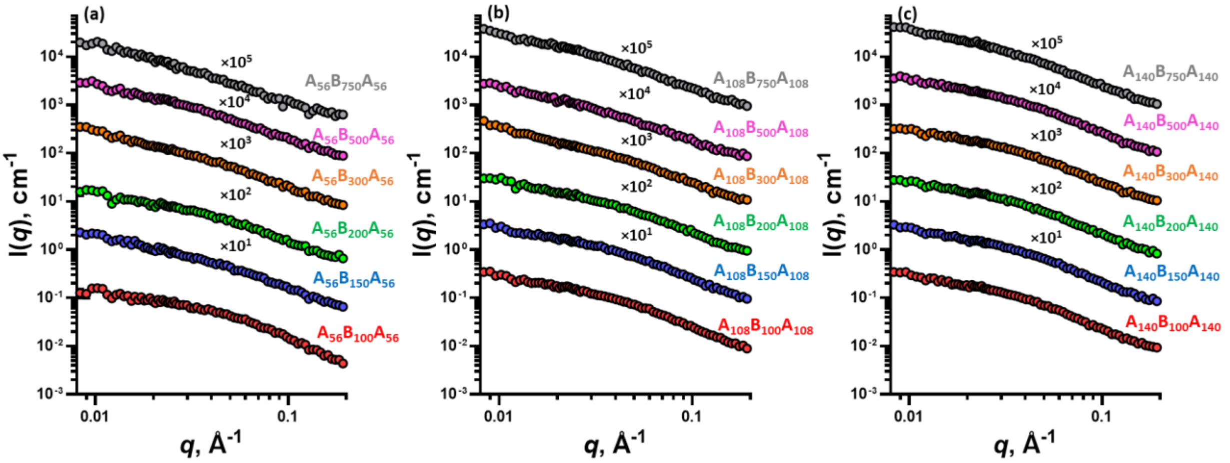

Figure S4. SAXS patterns of 1% w/w triblock copolymer solutions in MEK (symbols): (a) $A_{56}B_{100-750}A_{56}$, (b) $A_{108}B_{100-750}A_{108}$ and (c) $A_{140}B_{100-750}A_{140}$. Some patterns are shifted upward (the multiplication factors are indicated on the plots) to avoid overlap. Scattering patterns were collected using a Bruker AXS Nanostar instrument.

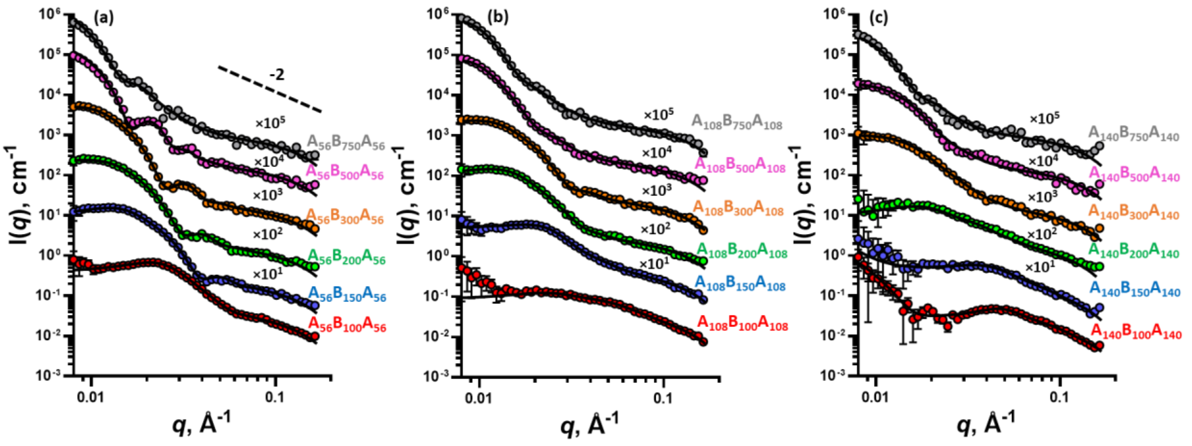

Figure S5. SAXS patterns of 1% w/w triblock copolymer aqueous dispersions (symbols) that are modelled with the two-population model (equations S13-15) (solid line): (a) $A_{56}B_{100-750}A_{56}$, (b) $A_{108}B_{100-750}A_{108}$ and (c) $A_{140}B_{100-750}A_{140}$. Some patterns are shifted upward (the multiplication factors are indicated on the plots) to avoid overlap. Scattering patterns were collected using a Bruker AXS Nanostar instrument.

Table S2. Results of SAXS analysis for P(AA-*st*-St) macro-CTA and P(AA-*st*-St)-*b*-PBA-*b*-P(AA-*st*-St) triblock copolymer 1% w/w aqueous dispersions where, R_{core} is the mean particle core radius and σ_{core} is its standard deviation, r_2 is the radius of the self-folded P(AA-*st*-St) chain on the surface of the particle (this value is fixed throughout the fitting at a value based upon the volume of the hard block), R_{PY} is the effective interparticle distance in the dispersion and the f_{PY} is the effective volume fraction corresponding to the hard sphere structure factor with Percus-Yevick (PY) closure.

Copolymer	R_{core} (Å)	σ_{core} (Å)	r_2 (Å)	R_{PY}	f_{PY}
$A_{56}A_{56}$	16	5	-	47	0.15
$A_{108}A_{108}$	16	7	-	53	0.16
$A_{140}A_{140}$	17	7	-	59	0.11
$A_{56}B_{100}A_{56}$	53	14	12	117	0.15
$A_{56}B_{150}A_{56}$	90	13	12	193	0.12
$A_{56}B_{200}A_{56}$	119	18	12	224	0.10
$A_{56}B_{300}A_{56}$	156	20	12	271	0.10
$A_{56}B_{500}A_{56}$	247	28	12	[a]	[a]
$A_{56}B_{750}A_{56}$	281	52	12	[a]	[a]
$A_{108}B_{100}A_{108}$	18	9	15	115	0.08
$A_{108}B_{150}A_{108}$	55	34	15	120	0.15
$A_{108}B_{200}A_{108}$	99	24	15	196	0.12
$A_{108}B_{300}A_{108}$	120	22	15	232	0.11
$A_{108}B_{500}A_{108}$	191	39	15	306	0.15
$A_{108}B_{750}A_{108}$	219	52	15	317	0.17
$A_{140}B_{100}A_{140}$	15	5	16	60	0.21
$A_{140}B_{150}A_{140}$	26	10	16	70	0.12
$A_{140}B_{200}A_{140}$	32	23	16	148	0.12
$A_{140}B_{300}A_{140}$	101	41	16	200	0.08
$A_{140}B_{500}A_{140}$	150	41	16	200	0.08
$A_{140}B_{750}A_{140}$	219	50	16	308	0.18

Note: [a] These parameters were excluded from the model as the scattering pattern was truncated by the *q*-range limit of the SAXS measurements.

Characterisation of the triblock copolymer films

Structural characterization of triblock copolymer films cast from an organic solvent

Solvent-cast films were drop-cast from a 40% w/w solution (approx. 100 µL) in MEK and formed transparent yellow films, where the yellowness of the film decreased as the triblock copolymer increased in molar mass. This reduction in yellow color is expected as the color is caused by the trithiocarbonate group present in the copolymers, the weight fraction of which decreases as the molar mass of the triblock copolymer increases. These films were cast onto dry-release film and left to dry under ambient conditions for 1 week. Where possible, the triblock film was removed from the silicone-coated dry-release film and transmission mode SAXS was performed on the free-standing film. Some triblock copolymer films were too soft and tacky to be removed without damage/distortion that may change their morphology. In these cases, SAXS was performed on the copolymer film whilst it was still attached to the dry-release film and a background scattering pattern of the dry-release film was subtracted from the combined scattering pattern.

It was expected that the hard and soft blocks within the triblock copolymer would undergo phase separation within the film and, since these two blocks have distinct scattering length densities, SAXS can be used to investigate the length scale of phase separation (Figure S6). A peak in scattering intensity was observed in the majority of the SAXS patterns of the copolymer films cast from MEK. The presence of this peak indicates that there is a structural order, which is likely to be associated with copolymer block phase separation occurring within the films.[1]

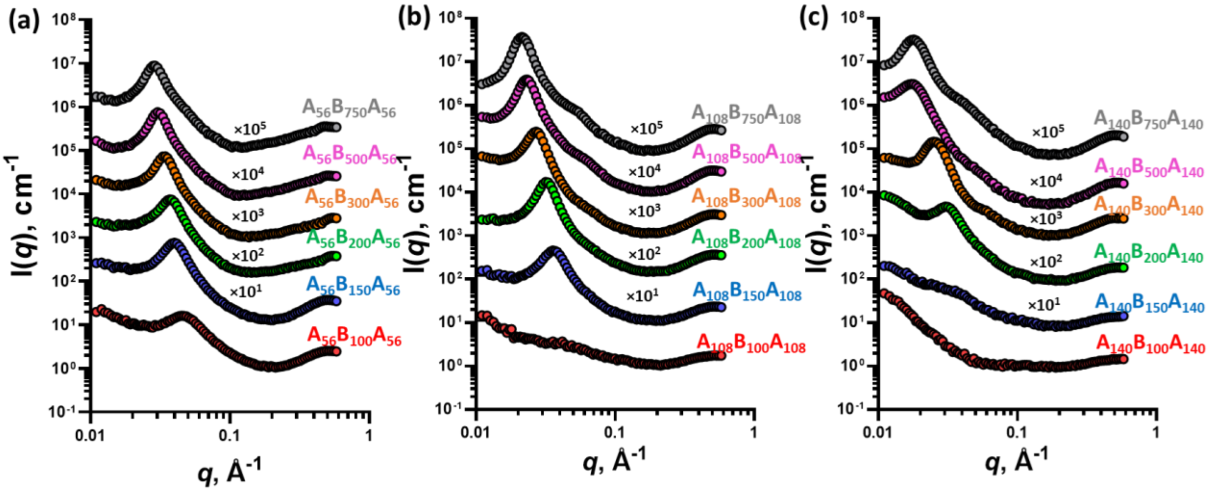

Figure S6. SAXS patterns of triblock copolymer films cast from a 40% w/w solution in MEK (symbols) for (a) $A_{56}B_{100\text{-}750}A_{56}$, (b) $A_{108}B_{100\text{-}750}A_{108}$ and (c) $A_{140}B_{100\text{-}750}A_{140}$ samples. Some patterns are shifted upwards (the multiplication factors are indicated on the plots) to avoid overlap. Scattering patterns were collected using a Xenocs Xeuss laboratory beamline.

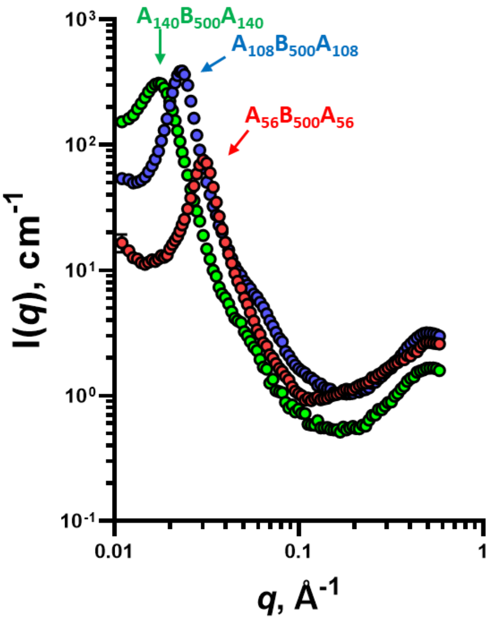

Figure S7. SAXS patterns of triblock copolymer films cast from a 40% w/w solution in MEK (symbols) for $A_{56}B_{500}A_{56}$, $A_{108}B_{500}A_{108}$, $A_{140}B_{500}A_{140}$ triblock copolymer films demonstrating how the length scale of the phase separation increases as the length of the hard block (A) increases. Scattering patterns were collected using a Xenocs Xeuss laboratory beamline.

Aside from the triblock copolymers with a short soft (B) block, the SAXS patterns of the solvent cast triblock copolymer films show a sharp peak in intensity suggesting that there is prominent phase separation within the films. The position of the primary peak (q^*) indicates the length scale of the phase separation (*d*-spacing) which can be calculated using the equation $d = \frac{2\pi}{q}$.[1] The SAXS patterns for the individual triblock series (*e.g.*, $A_{108}B_{100\text{-}750}A_{108}$) shows that the primary peak position shifts to a lower *q*-value as the DP of the soft BA block is increased (Figures S6, and Table S3) demonstrating that the length of the phase separation is increasing with the increasing soft block length (e.g. *d*-spacing of the $A_{108}B_{150\text{-}750}A_{108}$ series increases from 170 Å to 273 Å). Similarly, if the DP of soft block is kept constant when the hard block DP is increased the length of phase separation increases accordingly (Figure S7 and Table S3). Furthermore, increasing the overall triblock DP whilst maintaining a constant ratio of soft and hard units further increases the length of the phase separation (Table S3).

Table S3. Structural analysis by SAXS and AFM investigating the bulk and surface phase separation of the solvent-cast copolymer films, respectively, where q^* is position of the primary structural peak in the SAXS patterns, d_{SAXS} is the real space distance corresponding to q^* ($d_{SAXS} = 2\pi/q^*$), and d_{AFM} is the length scale of the phase separation measured by AFM (Figure S9). Structural morphology identified by AFM is given in the last column [Hex, BCC and Gyroid correspond to hexagonal-packed cylinders (space group P6/mm), body-centered cubic (space group Im3m) and gyroid cubic (space group Ia3d) structures, respectively]. The lattice period, a, was calculated using d_{SAXS}-spacing assuming that the main peak corresponds to 100, 110 and 211 reflections of Hex, BCC and Gyroid structures, respectively.

Triblock	q^* (Å^{-1})	d_{SAXS} (Å)	d_{AFM} (Å)	Structure
$A_{56}B_{100}A_{56}$	0.046	137	-	-
$A_{56}B_{150}A_{56}$	0.043	146	-	-
$A_{56}B_{200}A_{56}$	0.037	170	193	Hex, a = 196 Å
$A_{56}B_{300}A_{56}$	0.036	175	216	Hex, a = 202 Å
$A_{56}B_{500}A_{56}$	0.030	209	279	BCC, a = 296 Å
$A_{56}B_{750}A_{56}$	0.029	217	306	BCC, a = 307 Å
$A_{108}B_{100}A_{108}$	-	-	-	-
$A_{108}B_{150}A_{108}$	0.037	170	-	-
$A_{108}B_{200}A_{108}$	0.030	209	-	-
$A_{108}B_{300}A_{108}$	0.028	224	308	Gyroid, a = 549 Å
$A_{108}B_{500}A_{108}$	0.023	273	335	Hex, a = 315 Å
$A_{108}B_{750}A_{108}$	0.021	299	355	BCC, a = 423 Å
$A_{140}B_{100}A_{140}$	-	-	-	-
$A_{140}B_{150}A_{140}$	0.038	165	-	-
$A_{140}B_{200}A_{140}$	0.029	217	-	-
$A_{140}B_{300}A_{140}$	0.027	233	-	-
$A_{140}B_{500}A_{140}$	0.019	331	427	Gyroid, a = 811 Å
$A_{140}B_{750}A_{140}$	0.019	331	450	BCC, a = 468 Å

An additional broad diffuse peak is present in all the triblock copolymer films at q = 0.513 $Å^{-1}$ which equates to a distance of about 12.6 Å (Figures S6). This peak remains in the same position despite the variations in copolymer composition and is likely to be caused by the packing of the copolymer chains within the phase separated blocks. It was found that SAXS pattern of pure PBA polymer (Figure S10a) has a peak in a similar region to the one observed in the triblock copolymer films (Figures S6), whereas the pattern of P(AA-*st*-St) shows no such peak. This suggests that the peak observed in the triblock films is a result of structural packing of BA within the soft phase of the copolymer film (Figure S10b). Poly(butyl acrylate) has relatively long pendent groups that separate the copolymer backbones by a fixed distance from each other. A maximum packing distance, where the pendent groups are positioned without overlap/interdigitation, can be calculated as the product of the average C-C-C bond length (2.3 Å) and the number of bonds within the pendent group. The maximum packing distance was calculated to be 13.8 Å, which is comparable but larger than the measured separation (12.6 Å), indicating that the pendent chains are not in a fully extended conformation and possibly an interdigitation of the pendent groups takes place between adjacent PBA chains. A similar phenomenon has been observed in poly(behenyl methacrylate-*b*-benzyl methacrylate) [P(BeMA-*b*-BzMA)] nano-particles in mineral oil at 20 °C, where packing of behenyl groups within the PBeMA particle corona was observed in the SAXS patterns.[2]

Only one well-defined peak relating to the phase separated structure is observed in the SAXS patterns suggesting that the bulk structure of most of the compositions studied is not uniform. Attempts to obtain a more ordered structure by annealing film above the hard block T_g at 150 °C overnight only led to a slight sharpening of the primary peak. Thus, the structural morphology of the copolymer film could not be assessed purely through peak position analysis of the SAXS patterns. AFM was used to further investigate the phase separation of the solvent cast triblock copolymer films (Figure S8). Unlike transmission-mode SAXS which collects information

from the entire bulk of the film, AFM is only able to assess the surface structure. To obtain a high-quality image the sample surface has to be flat. This was a problem for the films that had a high hard to soft block ratio, since the difference between the Young's moduli of the comprising copolymer blocks would distort the film upon drying resulting in a brittle, uneven film surface. Therefore, AFM images were not collected for $A_{56}B_{100}A_{56}$, $A_{108}B_{100}A_{108}$, $A_{108}B_{200}A_{108}$, $A_{140}B_{100}A_{140}$, $A_{140}B_{200}A_{140}$ and $A_{140}B_{300}A_{140}$. Some of these copolymer films demonstrated a weak or no phase separation in the SAXS patterns (e.g., $A_{108}B_{100}A_{108}$, $A_{140}B_{100}A_{140}$, and $A_{140}B_{200}A_{140}$, Figure S6).

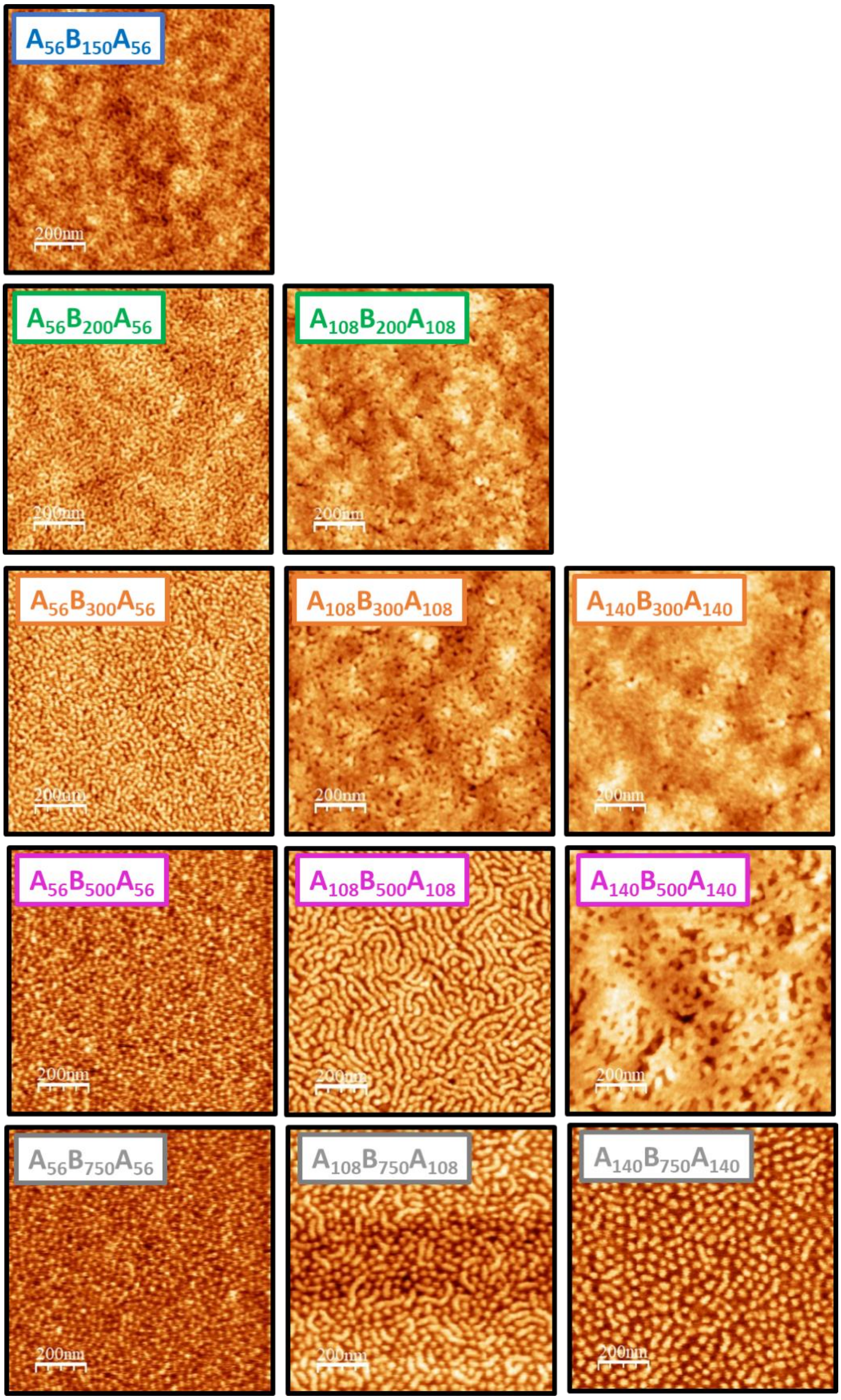

Figure S8. AFM height (topographic) images of triblock copolymer films cast from a 40% w/w solution in MEK measured using ScanAsyst® PeakForce® tapping mode (lighter color corresponds to higher structure relating to the hard phase), where the copolymer labels are shown on the respective images.

The AFM height images of the triblock films cast from MEK (Figure S8) indicate that there is phase separation visible on the surface of the films and that the structure and length of the phase separation varies with the copolymer composition. AFM images indicate that the length of the phase separation (Figure S8) increases as the hard block DP and the soft block DP increases, which is consistent with the SAXS results (Table S3). Furthermore, copolymers with a similar same hard-to-soft block ratio but with an increasing total DP (*e.g.*, $A_{56}B_{200}A_{56}$, $A_{108}B_{300}A_{108}$, and $A_{140}B_{500}A_{140}$) show an increase in the length of the phase separation, which agrees with the SAXS results.

Unlike SAXS, the surface structures observed by AFM clearly show that the relative ratios of the hard and soft components within the triblock copolymer have a large effect on the phase-separated structural morphology. Using the A_{108} triblock series as an example, $A_{108}B_{200}A_{108}$ has f_A of ~0.45 and possibly shows a biocontinuous structure (Figure S8). However, as f_A is reduced to ~0.36 for $A_{108}B_{300}A_{108}$ the bicontinuous structure becomes more apparent with the soft blocks (dark regions) emerging in the hard block matrix (light region). Upon a further reduction of f_A to ~0.25 ($A_{108}B_{500}A_{108}$), the regions of soft blocks form a continuous phase with cylinders formed from the hard block parallel to the surface. The final sample in this series, $A_{108}B_{750}A_{108}$, has an f_A of ~0.18 and the AFM image shows that the soft blocks are becoming dominant and forms a matrix around the hard blocks represented by cylindrical and, likely, spherical domains.

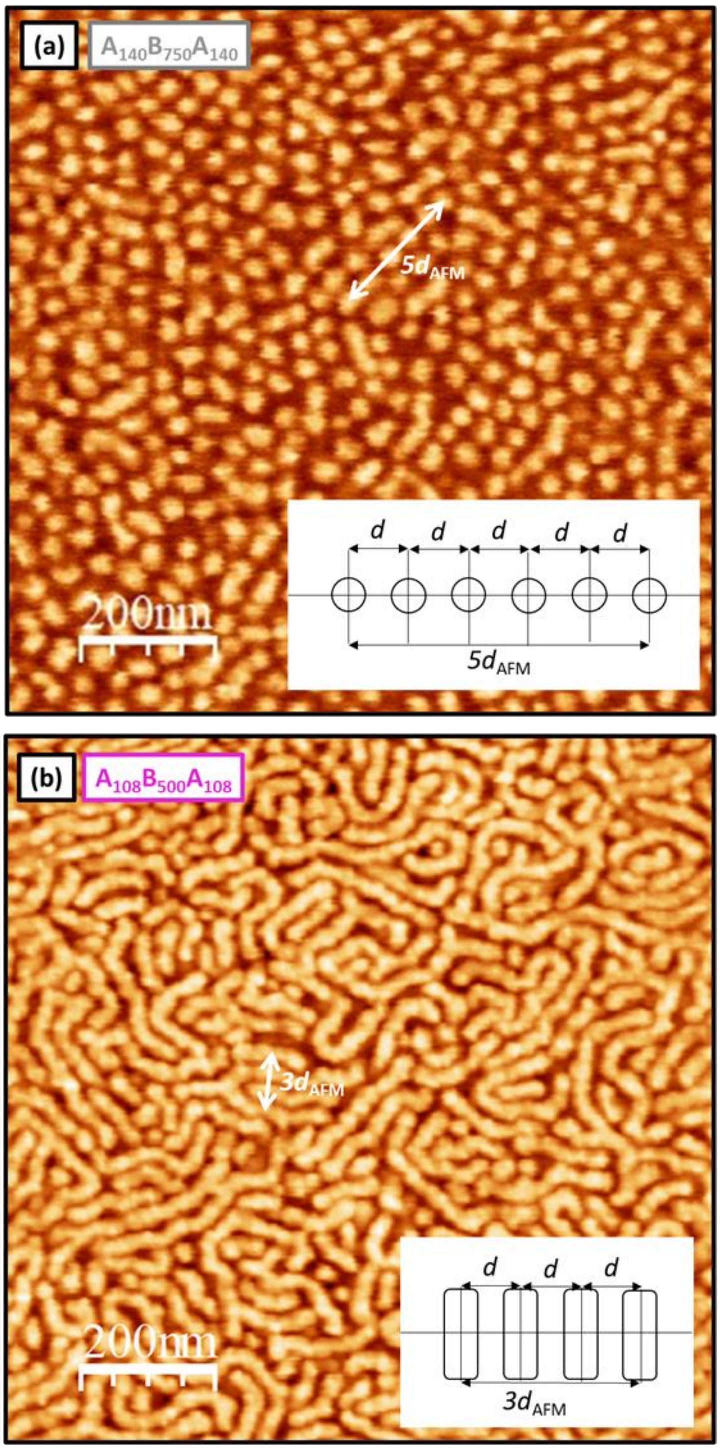

Figure S9. AFM height (topographic) image of an example triblock copolymer films (a) $A_{140}B_{750}B_{140}$ and (b) $A_{108}B_{500}B_{108}$ cast from a 40% w/w solution in MEK measured using ScanAsyst® PeakForce® tapping mode (lighter colour corresponds to higher structure relating to the hard phase), where the arrow demonstrates the measured distance, and the cartoon below demonstrates how d_{AFM} is calculated.

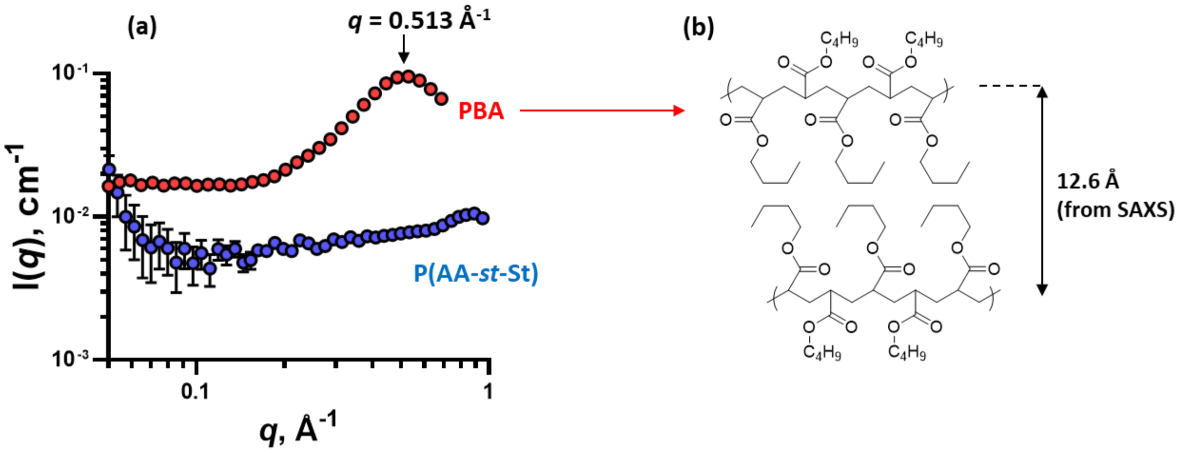

Figure S10. (a) SAXS patterns of solidified PBA and P(AA-*st*-St) polymers (symbols). (b) Diagram indicating the structural packing of BA units that induces a peak at q = 0.513 $Å^{-1}$ in the X-ray scattering pattern of PBA. Scattering patterns were collected using a Bruker AXS Nanostar instrument.

Diffraction peak indexing of SAXS patterns of dried P(AA-*st*-St)-*b*-PBA-*b*-P(AA-*st*-St) films cast from an organic solvent

A comparison of the length of phase separation measured from SAXS (d_{SAXS}) and from AFM (d_{AFM}) data (Table S3) suggests that d_{SAXS} are systematically smaller than d_{AFM}. This apparent inconsistency is because the distances measured *via* SAXS are *d*-spacings corresponding to the crystallographic planes formed by objects structurally-ordered in three-dimensional space, whereas AFM measures distances between objects exposed to a two-dimensional surface which could correspond to a particular crystallographic plane.[1] Following the phase diagram of diblock copolymers[3] and AFM results (Figure S8 and Table S3), diffraction peaks observed in the SAXS patterns of P(AA-*st*-St)-*b*-PBA-*b*-P(AA-*st*-St) films (Figure S6) could be indexed

by Miller indexes corresponding to appropriate crystallographic phases (Figure S11 and Table S3).

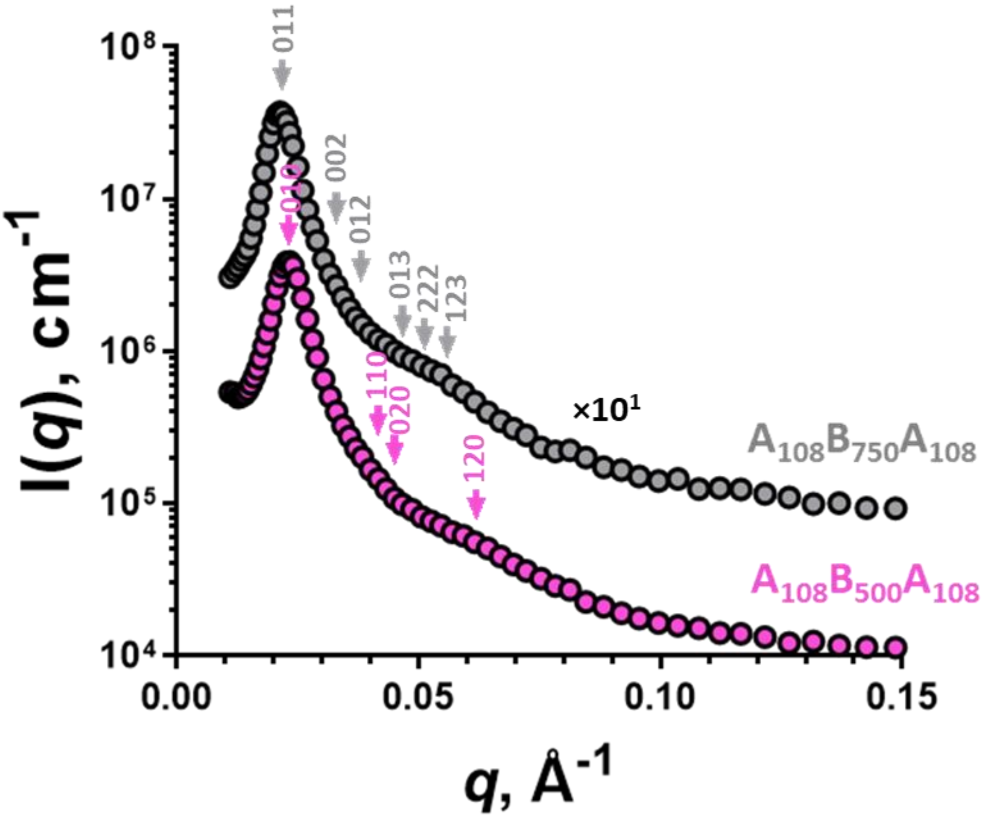

Figure S11. SAXS patterns of $A_{108}B_{750}A_{108}$ and $A_{108}B_{500}A_{108}$ triblock copolymer films cast from a 40% w/w solution in MEK (grey and pink symbols, respectively), where the arrows indicate the theoretical positions of the diffraction peaks for a BCC structure (grey arrows) and a hexagonal structure (pink arrows). The $A_{108}B_{750}A_{108}$ pattern is shifted upwards (the multiplication factor is indicated on the plots) to avoid overlap. Scattering patterns were collected using a Xenocs Xeuss instrument.

For example, both a theoretical diblock copolymer phase diagram[3] and the AFM results (Figure S8) suggest that the scattering patterns of $A_{108}B_{750}A_{108}$ and $A_{108}B_{500}A_{108}$, demonstrating a similar *q*-value for the main peak position (Table S3), should correspond mainly to BCC structure and hexagonally packed cylinders, respectively. A BCC structure should have a first few characteristic diffraction peaks with Miller indices, *hkl*, of 011, 002, 112, 013, 222, and

123, which relate to (q^*) with ratios (q/q^*) of √1, √2, √3, √5, √6 and √7, respectively. Whereas the Miller indices of a hexagonally packed cylinder structure are 010, 110, 020 and 120, which relate to the main peak position with ratios of √1, √3, √4, and √7, respectively. Although the phase separated structures within the triblock films are not ordered enough to display sharp diffraction peaks relating to the expected crystal structure reflections, there are broad diffuse peaks in the regions corresponding to the location of the expected diffraction peaks (Figure S11). Assuming that $A_{108}B_{750}A_{108}$ has a BCC structure, the distance, d_{011}, between the {011} crystallographic planes, measured from the main peak position of the $A_{108}B_{750}A_{108}$ SAXS pattern, can be used to calculate the cubic lattice period, a_{BCC}, according to:

$$\frac{1}{d_{hkl}^2} = \frac{h^2 + k^2 + l^2}{a_{\mathrm{BCC}}^2} \tag{S1}$$

The shortest distance between nearest spheres forming a BCC structure could be calculated as a half of the cube diagonal length: $a = a_{\mathrm{BCC}}\sqrt{3}/2$ (Figure S12). Since d_{011} of $A_{108}B_{750}A_{108}$ should be equal to 273 Å (Table S3), this converts into the BCC lattice period of a_{BCC} = 386 Å and the shortest inter-particle distance of a = 334 Å. This calculated values representing distances between neighbouring particles in a BCC structure are reasonably consistent with the average distance between particles measured by AFM (d_{AFM} = 353 Å, Table S3) and demonstrates the consistency between the two structural characterisation methods used within this paper. A similar calculation can be performed on copolymers that have a separation in the bulk represented by a hexagonally packed cylinders (*e.g.*, $A_{108}B_{500}A_{108}$). Inter-plane distances of a hexagonal structure relate to the lattice periods as:

$$\frac{1}{d_{hkl}^2} = \frac{4}{3}\frac{h^2 + hk + k^2}{a_{\mathrm{hex}}^2} + \frac{l^2}{c_{\mathrm{hex}}^2} \tag{S2}$$

Assuming that dried $A_{108}B_{500}A_{108}$ copolymers form hexagonally packed cylinders, the main peak observed in the SAXS pattern should correspond to an inter-plane distance, d_{010}, of 273 Å (Figure S11). According to eq S2 this value converts to a_{hex} = 315 Å corresponding to an inter-cylinder distance; again, this is similar to the distance measured by AFM (d_{AFM} = 335 Å, Table S3).

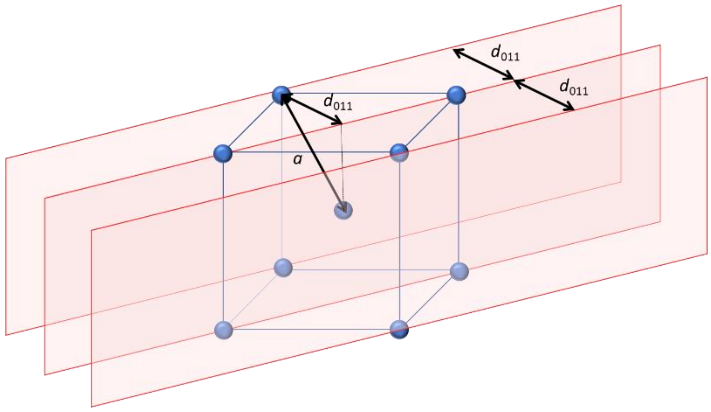

Figure S12. A cartoon depiction of a BCC crystal structure showing the distances between the {011} crystallographic planes, d_{011}, and the shortest distance between spheres packed in a BCC structure, a.

Structural characterization of triblock copolymer films cast from aqueous dispersions

The films were prepared by drop-casting 20% w/w (approx. 100 μL) triblock copolymer dispersions onto dry release film in the same way to the films cast from MEK. Transmission-mode SAXS was used to investigate the size and structure of the phase separation within the bulk of the film and SAXS patterns were collected for all the triblock copolymer films (Figure S13).

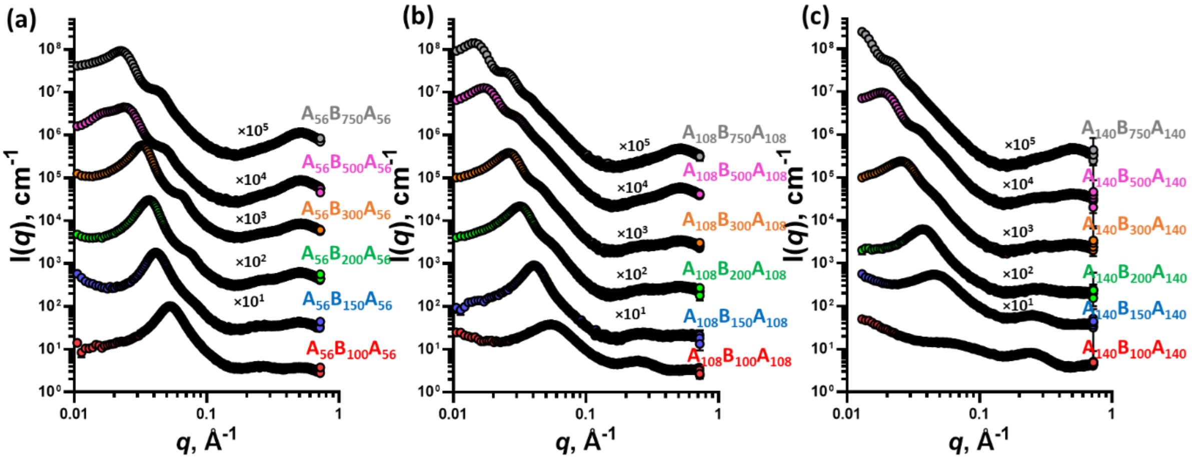

Figure S13. SAXS patterns of triblock copolymer films cast from a 20% w/w aqueous dispersion (symbols) for (a) $A_{56}B_{100\text{-}750}A_{56}$, (b) $A_{108}B_{100\text{-}750}A_{108}$ and (c) $A_{140}B_{100\text{-}750}A_{140}$ compositions. Some patterns are shifted upwards (the multiplication factors are indicated on the plots) to avoid overlap. Scattering patterns were collected using a Xenocs Xeuss laboratory beamline.

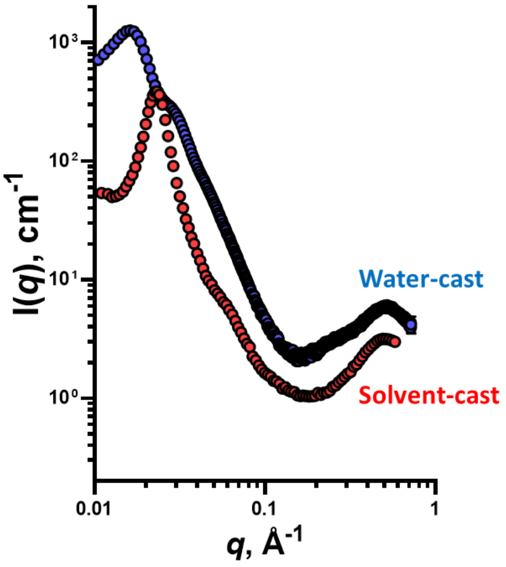

Figure S14. SAXS patterns of $A_{108}B_{500}A_{108}$ copolymer films cast from either a 20% w/w aqueous dispersion (blue symbols), or a 40% w/w solution in MEK (red symbols). Scattering patterns were collected using a Xenocs Xeuss instrument.

The length scale of phase separation observed by SAXS for the films cast from water (Table S4, Figure S13) shows a similar trend as for the films cast from MEK (Table S3 and Figure S6), where the size of the phase separation increases as the length of either the soft or hard blocks, or the total triblock length, is increased. Despite these similarities between the aqueous cast films and the solvent cast films, the general structure of the phase separation is different as is demonstrated by the different shapes of the scattering patterns (Figure S14). Generally, the water-cast triblock copolymer films show phase separation on a longer length scale. For example, the primary peak for each of the triblock copolymers appears at a lower *q*-value when cast from water rather than MEK (e.g., the peak for the $A_{108}B_{500}A_{108}$ films appears at 0.0154 $Å^{-1}$ in water and 0.0235 $Å^{-1}$ in MEK). Furthermore, clear secondary and tertiary scattering intensity peaks are observed in some of the SAXS patterns of the water-cast films (e.g. peaks at approx. 0.03 and 0.04 $Å^{-1}$ for $A_{108}B_{750}A_{108}$, Figure S13b). The presence of these extra peaks

suggests that the phase separation in the bulk of the film is better defined than in the solvent-cast films.

In addition to the high intensity peaks present at low *q*-values, there are a few peaks present in the high *q* region that are a result of smaller, well-ordered structures within the film. Similar to the solvent-cast films, the SAXS patterns of water-cast films contain a peak at q = 0.513 Å^{-1} that is likely to be a result of the PBA packing within the soft domains of the formed structures. Another peak at q ~ 0.25 Å^{-1}, observed only for the water-cast films (Figure S13), could be a result of the structure formed by packing of the statistical A block of a particulate nature proposed for the triblock copolymer particles (Figure 5). The relative intensities of the two peaks vary with the copolymer composition, with the peak at q = 0.513 Å^{-1} increasing in intensity as the fraction of the soft block increases while the peak at q ~ 0.25 Å^{-1} becomes more prominent when the fraction of the hard block increases (Figure S13). Thus, the relative intensities of these two peaks with respect to the copolymer composition further justify the cause of these structural peaks.

The SAXS patterns of the majority of the water-cast triblock copolymer films ($A_{56}B_{100\text{-}300}A_{56}$, $A_{108}B_{100\text{-}300}A_{108}$, and $A_{140}B_{100\text{-}300}A_{140}$) were fit using a sphere form factor (equations S9-S12) combined with a structure factor of interacting hard spheres (eq S7) (Figure S15). Comparison between the modelled spherical domain radius within the triblock copolymer films and the core radius of the particles in the aqueous dispersion showed that, generally, the domain size (Table S4) is significantly smaller than the respective particle size (Table 3). This suggests that once the water has evaporated some of the PBA chains are no longer present in the well-defined core that is observed in the SAXS patterns and become part of the surrounding matrix (Figure 9a). However, this reduction in the domain size is not observed for the triblock copolymers with the largest hard (A) block length and a relatively small BA block (*i.e.*, $A_{140}B_{100\text{-}200}A_{140}$). This suggests the releasing of PBA chains from the core only occurs when the copolymer

composition is weighted towards the soft block. Furthermore, it is only when the PBA block becomes larger (greater DP) than the total stabilizer block ($A_{140}B_{300}A_{140}$) that a reduction in the domain size upon drying is observed. As the PBA core is highly hydrophobic, it is unlikely that the reduction in domain size is due to loss of water from the particle core. Additionally, the core domain size appears to be dominated largely by the size of the PBA block and is fairly independent of the length of the hard block. For example, $A_{56}B_{300}A_{56}$, $A_{108}B_{300}A_{108}$, and $A_{140}B_{300}A_{140}$, have a hydrophobic domain size of 54 Å, 53 Å and 58 Å, respectively.

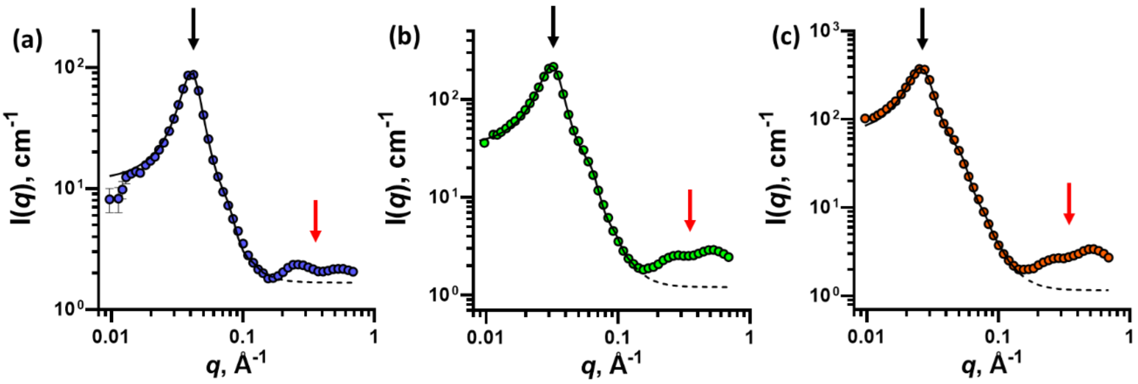

Figure S15. SAXS patterns of triblock copolymer films cast from a 20% w/w aqueous dispersion (symbols) fitted using a sphere form factor combined with a hard sphere structure factor (black line), where (a) is $A_{108}B_{150}A_{108}$, (b) is $A_{108}B_{200}A_{108}$, and (c) is $A_{108}B_{300}A_{108}$. The black arrow indicated where there is a good correlation between the experimental scattering pattern and the structural model and the red arrow indicates the region where there is a deviation from the model due to additional structures that have not been accounted for (*e.g.* the packing of particles formed by the hard (A) block and the packing of the BA chains). The scattering patterns were collected using a Xenocs Xeuss laboratory beamline.

Table S4. Results of SAXS analysis of the bulk phase separation within the water-cast P(AA-*st*-St)-*b*-PBA-*b*-P(AA-*st*-St) triblock copolymer films, where q^* is the position of the primary structural peak in the SAXS pattern, d is the associated distance in real space ($d = 2\pi/q^*$), R_s is the mean radius of the spherical BA domain within the film and σ_s is its standard deviation, R_{PY} is the interdomain radius in the film matrix and the f_{PY} is the effective volume fraction corresponding to the hard sphere structure factor with Percus-Yevick (PY) closure.

Triblock	q^* (Å^{-1})	d (Å)	R_s (Å)	σ_s (Å)	R_{PY} (Å)	f_{PY}
$A_{56}B_{100}A_{56}$	0.053	119	34	6	58	0.34
$A_{56}B_{150}A_{56}$	0.041	153	44	10	76	0.36
$A_{56}B_{200}A_{56}$	0.04	157	52	18	86	0.37
$A_{56}B_{300}A_{56}$	0.031	203	54	33	96	0.37
$A_{56}B_{500}A_{56}$	0.024	262	-	-	-	-
$A_{56}B_{750}A_{56}$	0.023	273	-	-	-	-
$A_{108}B_{100}A_{108}$	0.053	119	30	4	51	0.22
$A_{108}B_{150}A_{108}$	0.041	153	45	11	76	0.34
$A_{108}B_{200}A_{108}$	0.030	209	50	19	96	0.31
$A_{108}B_{300}A_{108}$	0.029	217	53	27	116	0.29
$A_{108}B_{500}A_{108}$	0.019	331	-	-	-	-
$A_{108}B_{750}A_{108}$	0.015	419	-	-	-	-
$A_{140}B_{100}A_{140}$	0.067	94	22	1	43	0.11
$A_{140}B_{150}A_{140}$	0.045	140	32	10	58	0.19
$A_{140}B_{200}A_{140}$	0.036	175	45	13	78	0.27
$A_{140}B_{300}A_{140}$	0.028	224	58	22	113	0.25
$A_{140}B_{500}A_{140}$	0.019	331	-	-	-	-
$A_{140}B_{750}A_{140}$	-	-	-	-	-	-

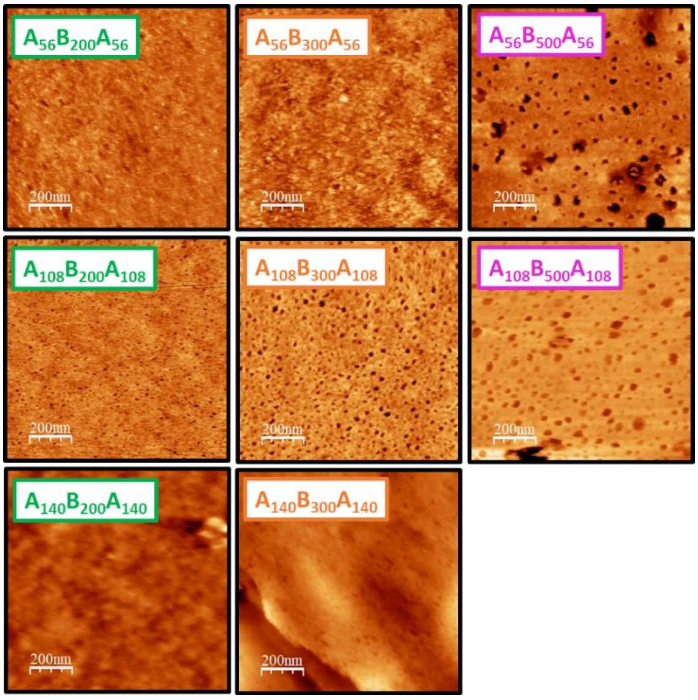

Figure S16. AFM height (topographic) images of triblock copolymer films cast from a 20% w/w aqueous solution measured using ScanAsyst® PeakForce® tapping mode, where the copolymer labels are shown on the respective images.

Mechanical characterization of the triblock copolymer films

Table S5. Tensile extension results where ▲ indicates that the triblock film was too brittle to test and ● indicates that the film was too soft to test.

	Solvent		Aqueous	
Triblock	Modulus (MPa)	Extension-to-break (%)	Modulus (MPa)	Extension-to-break (%)
$A_{56}B_{100}A_{56}$	▲	▲	▲	▲
$A_{56}B_{150}A_{56}$	83.7 ± 16.6	85.2 ± 34.9	▲	▲
$A_{56}B_{200}A_{56}$	35.6 ± 9.2	99.5 ± 39.4	89.8 ± 31.4	17.3 ± 6.9
$A_{56}B_{300}A_{56}$	0.45 ± 0.13	206.1 ± 40.2	63.3 ± 9.0	26.5 ± 4.1
$A_{56}B_{500}A_{56}$	●	●	116.9 ± 20.1	17.1 ± 4.6
$A_{56}B_{750}A_{56}$	●	●	●	●
$A_{108}B_{100}A_{108}$	▲	▲	▲	▲
$A_{108}B_{150}A_{108}$	▲	▲	▲	▲
$A_{108}B_{200}A_{108}$	▲	▲	▲	▲
$A_{108}B_{300}A_{108}$	171.7 ± 34.6	13.3 ± 3.7	238.3 ± 45.9	10.5 ± 5.4
$A_{108}B_{500}A_{108}$	1.19 ± 0.21	176.8 ± 20.9	123.2 ± 11.1	9.4 ± 1.0
$A_{108}B_{750}A_{108}$	●	●	●	●
$A_{140}B_{100}A_{140}$	▲	▲	▲	▲
$A_{140}B_{150}A_{140}$	▲	▲	▲	▲
$A_{140}B_{200}A_{140}$	▲	▲	▲	▲
$A_{140}B_{300}A_{140}$	259.4 ± 102.8	10.4 ± 4.6	▲	▲
$A_{140}B_{500}A_{140}$	89.8 ± 23.3	27.8 ± 10.9	254.5 ± 180.5	11.2 ± 9.6
$A_{140}B_{750}A_{140}$	●	●	188.1 ± 50.5	14.5 ± 6.1

SAXS Structural Models

Intensity equation

The intensity of the X-rays scattered by a particle dispersion, $I(q)$, is expressed as:

$$I(q) = N \cdot S(q) \cdot \int_0^\infty \ldots \int_0^\infty F(q, r_1, \ldots, r_k) \cdot \Psi(r_1, \ldots, r_k) dr_1 \ldots dr_k \qquad \text{(S3)}$$

where $F(q, r_1, \ldots, r_k)$ is the particle form factor defined by a k number of structural parameters, $\Psi(r_1, \ldots, r_k)$ is a function describing dispersity of these parameters, $S(q)$ is the structure factor describing particle interactions in the dispersion and N is the number density per unit sample volume defined as:

$$N = \frac{\phi}{\int_0^\infty \ldots \int_0^\infty V(r_1, \ldots, r_k) \cdot \Psi(r_1, \ldots, r_k) dr_1 \ldots dr_k} \qquad \text{(S4)}$$

where ϕ is the total particle volume fraction and $V(r_1, \ldots, r_k)$ is the particle volume.

Intensity equation with linear background

A plateau in intensity at high q was observed in the majority of the scattering patterns after subtraction of background scattering originating from the solvent and the sample holder (*i.e.*, glass capillary). This scattering signal is likely to be associated with fluctuations in scattering length density across the particles caused by random distribution of monomer units in the self-assembled copolymers. In order to account for these fluctuations a linear fitting parameter (C_1) that is independent of the scattering vector q was added to the equation describing the intensity of scattered X-rays (eq. S3):

$$I(q) = N \cdot S(q) \cdot \int_0^\infty \ldots \int_0^\infty F(q, r_1, \ldots, r_k) \cdot \Psi(r_1, \ldots, r_k) dr_1 \ldots dr_k + C_1 \qquad \text{(S5)}$$

Distribution function of the structural model parameters

Only dispersity of particle radius, r, expressed as a Gaussian distribution, is considered for the structural models using eq. S3 (or equation S5):

$$\Psi(r) = \frac{1}{\sqrt{2\pi\sigma_R^2}} e^{\frac{(r-R)^2}{2\sigma_R^2}} \tag{S6}$$

where R is the mean particle radius and σ_R is its standard deviation. All other fitting parameters describing the structural models where considered to be monodisperse (their distribution functions correspond to Dirac's delta function).

Hard sphere structure factor

A hard sphere structure factor solved using the Percus-Yevick closure relation:[4]

$$S(q) = S_{\mathrm{PY}}(q, R_{\mathrm{PY}}, f_{\mathrm{PY}}) \tag{S7}$$

where R_{PY} is an effective interparticle correlation radius and f_{PY} is an effective volume fraction, has been incorporated into eq. S3 to account for long range interactions between the particles.[5]

Form factor for a Gaussian chain

A polymer molecule in theta solvent conditions behave as a Gaussian chain with a radius of gyration, R_{g}. In this case form factor of the polymer is described by a Debye function:

$$F_{\mathrm{chain}}(x) = \frac{2(e^{-x}-1+x)}{x^2} \tag{S2}$$

where $x = (qR_g)^2$.

Sphere model

The self-assembled statistical copolymers studied in this work can be described as homogenous spherical particles. Thus, the form factor for eq. S3 (or equations S5) can be defined as:[6,7]

$$F_s(q,r) = N_{\mathrm{agg}}(r)\beta_s{}^2 \cdot A_s{}^2(q \cdot r) \quad \text{(S3)}$$

where r is the spherical particle radius, β_s is the scattering length density (SLD) contrast of the particle defined as $\beta_s = V(r)(\xi_{\mathrm{cop}} - \xi_{\mathrm{sol}})$. Herein $V(r) = \frac{4\pi r^3}{3}$, and ξ_A and ξ_{sol} represent the SLDs of the copolymer and background solvent, respectively. ξ_A in this case represents the averaged SLD of the statistical copolymer calculated as:

$$\xi_A = \xi_{\mathrm{St}} \cdot v_{\mathrm{St}} + \xi_{\mathrm{AA}} \cdot v_{\mathrm{AA}} \quad \text{(S4)}$$

where $\xi_{\mathrm{St}} = 9.45 \times 10^{-10}$ cm^{-2} and $\xi_{\mathrm{AA}} = 12.62 \times 10^{-10}$ cm^{-2} are the SLDs of styrene and acrylic acid units, respectively, and v_{St} and v_{AA} are the volume fractions of styrene and acrylic acid units in a polymer chain, respectively. $A_s(qr)$ corresponds to a function describing the normalized sphere form factor amplitude:

$$A_s(qr) = \frac{3[sin(qr) - qr \cdot cos(qr)]}{(qr)^3} \quad \text{(S5)}$$

Additionally, the molecule aggregation number can be calculated from the model parameters:

$$N_{\mathrm{agg}} = (1 - x_{\mathrm{sol}})\frac{V(r)}{V_A} \quad \text{(S6)}$$

where x_{sol} is the volume fraction of solvent within the sphere and V_A is the volume of a single copolymer molecule.

Two-population model

Generally, the intensity of the X-rays scattered by a particle dispersion, $I(q)$, is defined by eq S3. However, in a case of core-shell spherical particles with a particulate shell (Figure S15) eq. S3 should be altered to account for the additional surface structure:

$$I(q) = S_1(q) \cdot N_1 \cdot \int_0^\infty F_1(q, r_1) \cdot \Psi(r_1) dr_1 + S_2(q) \cdot N_2 \cdot \int_0^\infty F_2(q, r_2) \cdot \Psi(r_2) dr_2 \quad \text{(S7)}$$

where the subscript 1 and 2 denotes the two populations of spherical particles, where the population 1 is assigned to the core-shell particles formed by triblock copolymers, and the population 2 to the particles comprising the triblock copolymer particle shell (Figure S17).

In this case the population 1 is described as a core-shell particle and therefore $F_1(q, r_1)$ can be defined as:

$$F_1(q, r_1, \Delta r, \xi_{\text{core}}, \xi_{\text{shell}}) = {A_1}^2(q, r_1, \Delta r, \xi_{\text{core}}, \xi_{\text{shell}}) \quad \text{(S8)}$$

where the form factor amplitude is defined as:[6,7]

$$A_1(q, r_1, \Delta r, \xi_{\text{core}}, \xi_{\text{shell}}) = (\xi_{\text{core}} - \xi_{\text{sol}}) V_{\text{part}} A_s(q \cdot r_1) + (\xi_{\text{core}} - \xi_{\text{shell}}) V_{\text{core}} A_s[q \cdot (r_1 - \Delta r)] \quad \text{(S15)}$$

where ξ_{shell} and ξ_{core} are the SLDs of the shell and core, respectively (ξ_{core} is equal to the SLD of PBA, which is ξ_{BA} = 10.07 × 10^{-10} cm^{-2}, and ξ_{shell} = 10.55 × 10^{-10} cm^{-2}, calculated using eq S10). r_1 and $V_{\text{part}} = \frac{4\pi r_1^3}{3}$, are the radius and volume of the entire particle, and Δr is the shell thickness. $V_{\text{core}} = \frac{4\pi(r_1 - \Delta r)^3}{3}$ is volume of the particle core.

Whereas the population 2 is described as a simple sphere and, therefore, $F_2(q, r_2)$ in eq S13 can be described using equations S9, S10, and S11 where it should be assumed that $r = r_2$.

Additionally, the structure factors S_1 and S_2 are described using the hard-sphere structure factor solved using the Percus-Yevick closure relation (eq S7).

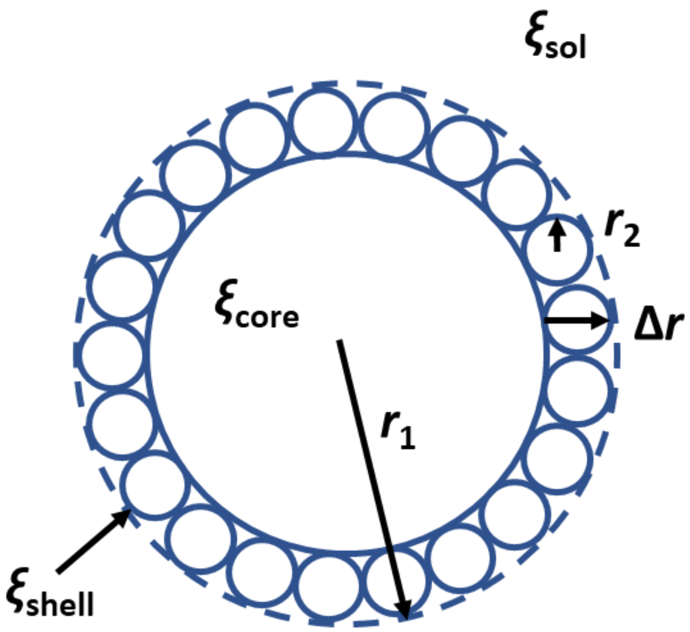

Figure S17. Schematic diagram of a core-shell particle formed by self-assembled P(AA-*st*-St)-*b*-PBA-*b*-P(AA-*st*-St) triblock copolymers where the core is composed of the PBA blocks and the shell is composed of particles formed by "self-folded" P(AA-*st*-St) statistical copolymer blocks.

Gaussian distribution

The polydispersity of the particle radius, expressed as a Gaussian distribution, is considered for the structural model (eq S13):

$$\Psi(r) = \frac{1}{\sqrt{2\pi\sigma_R^2}} e^{-\frac{(r-R)^2}{2\sigma_R^2}} \quad \text{(S16)}$$

where R is the mean particle radius and σ_R is its standard deviation.

References

(1) Mykhaylyk, T. A.; Mykhaylyk, O. O.; Collins, S.; Hamley, I. W. Ordered Structures and Phase Transitions in Mixtures of a Polystyrene/Polyisoprene Block Copolymer with the Corresponding Homopolymers in Thin Films and in Bulk. *Macromolecules* **2004**, *37* (9), 3369–3377.

(2) Derry, M. J.; Mykhaylyk, O. O.; Ryan, A. J.; Armes, S. P. Thermoreversible Crystallization-Driven Aggregation of Diblock Copolymer Nanoparticles in Mineral Oil. *Chem. Sci.* **2018**, *9* (17), 4071–4082.

(3) Mai, Y.; Eisenberg, A. Self-Assembly of Block Copolymers. *Chem. Soc. Rev.* **2012**, *41* (18), 5969–5985.

(4) Percus, J. K.; Yevick, G. J. Analysis of Classical Statistical Mechanics by Means of Collective Coordinates. *Phys. Rev.* **1958**, *110* (1), 1–13.

(5) Neal, T. J.; Beattie, D. L.; Byard, S. J.; Smith, G. N.; Murray, M. W.; Williams, N. S. J.; Emmett, S. N.; Armes, S. P.; Spain, S. G.; Mykhaylyk, O. O. Self-Assembly of Amphiphilic Statistical Copolymers and Their Aqueous Rheological Properties. *Macromolecules* **2018**, *51* (4), 1474–1487.

(6) Pedersen, J. S. Analysis of Small-Angle Scattering Data from Colloids and Polymer Solutions: Modeling and Least-Squares Fitting. *Adv. Colloid Interface Sci.* **1997**, *70*, 171–210.

(7) Pedersen, J. S. Form Factors of Block Copolymer Micelles with Spherical, Ellipsoidal and Cylindrical Cores. *J. Appl. Crystallogr.* **2000**, *33*, 637–640.
